# On the Distribution of the Information Density of Gaussian Random Vectors: Explicit Formulas and Tight Approximations

**DOI:** 10.3390/e24070924

**Published:** 2022-07-02

**Authors:** Jonathan E. W. Huffmann, Martin Mittelbach

**Affiliations:** 1 Lehrstuhl für Theoretische Informationstechnik, Fakultät für Elektrotechnik und Informationstechnik, Technische Universität München, 80290 München, Germany; jonathan.huffmann@tum.de; 2 Lehrstuhl für Theoretische Nachrichtentechnik, Fakultät für Elektrotechnik und Informationstechnik, Technische Universität Dresden, 01062 Dresden, Germany

**Keywords:** information density, information spectrum, probability density function, cumulative distribution function, central moments, Gaussian random vector, canonical correlation analysis

## Abstract

Based on the canonical correlation analysis, we derive series representations of the probability density function (PDF) and the cumulative distribution function (CDF) of the information density of arbitrary Gaussian random vectors as well as a general formula to calculate the central moments. Using the general results, we give closed-form expressions of the PDF and CDF and explicit formulas of the central moments for important special cases. Furthermore, we derive recurrence formulas and tight approximations of the general series representations, which allow efficient numerical calculations with an arbitrarily high accuracy as demonstrated with an implementation in Python publicly available on GitLab. Finally, we discuss the (in)validity of Gaussian approximations of the information density.

## 1. Introduction and Main Theorems

Let ξ and η be arbitrary random variables on an abstract probability space (Ω,F,P) such that the joint distribution $P_{\xi \eta }$ is absolutely continuous w. r. t. the product $P_{\xi }⊗P_{\eta }$ of the marginal distributions $P_{\xi }$ and $P_{\eta }$. If $\frac{\;dP_{\xi \eta }}{\;dP_{\xi }⊗P\eta }$ denotes the Radon–Nikodym derivative of $P_{\xi \eta }$ w. r. t. $P_{\xi }⊗P_{\eta }$, then

$$\begin{array}{c} i\left(\xi ;\eta \right)=\log\left(\frac{\;dP_{\xi \eta }}{\;dP_{\xi }⊗P_{\eta }}\left(\xi ,\eta \right)\right) \end{array}$$

is called the information density of ξ and η. The expectation E(i(ξ;η))=I(ξ;η) of the information density, called mutual information, plays a key role in characterizing the asymptotic channel coding performance in terms of channel capacity. The non-asymptotic performance, however, is determined by the higher-order moments of the information density and its probability distribution. Achievability and converse bounds that allow a finite blocklength analysis of the optimum channel coding rate are closely related to the distribution function of the information density, also called information spectrum by Han and Verdú [1,2]. Moreover, based on the variance of the information density tight second-order finite blocklength approximations of the optimum code rate can be derived for various important channel models. First work on a non-asymptotic information theoretic analysis was already published in the early years of information theory by Shannon [3], Dobrushin [4], and Strassen [5], among others. Due to the seminal work of Polyanskiy et al. [6], considerable progress has been made in this area. The results of [6] on the one hand and the requirements of current and future wireless networks regarding latency and reliability on the other hand stimulated a significant new interest in this type of analysis (Durisi et al. [7]).

The information density i(ξ;η) in the case when ξ and η are jointly Gaussian is of special interest due to the prominent role of the Gaussian distribution. Let $\xi =(\xi_{1},\xi_{2},\ldots ,\xi_{p})$ and $\eta =(\eta_{1},\eta_{2},\ldots ,\eta_{q})$ be real-valued random vectors with nonsingular covariance matrices $R_{\xi }$ and $R_{\eta }$ and cross-covariance matrix $R_{\xi rvY}$ with rank $r=\mathrm{rank}(R_{\xi \eta })$. (For notational convenience, we write vectors as row vectors. However, in expressions where matrix or vector multiplications occur, we consider all vectors as column vectors.) Without loss of generality for the subsequent results, we assume the expectation of all random variables to be zero. If $\left(\xi_{1},\xi_{2},\ldots ,\xi_{p},\eta_{1},\eta_{2},\ldots ,\eta_{q}\right)$ is a Gaussian random vector, then Pinsker [8], Ch. 9.6 has shown that the distribution of the information density i(ξ;η) coincides with the distribution of the random variable

(1) $$\begin{array}{cc} \nu & =\frac{1}{2}\sum_{i=1}^{r}\varrho_{i}\left({\tilde{\xi }}_{i}^{2}-{\tilde{\eta }}_{i}^{2}\right)+I\left(\xi ;\eta \right). \end{array}$$

In this representation ${\tilde{\xi }}_{1},{\tilde{\xi }}_{2},\ldots ,{\tilde{\xi }}_{r},{\tilde{\eta }}_{1},{\tilde{\eta }}_{2},$…$,{\tilde{\eta }}_{r}$ are independent and identically distributed (i.i.d.) Gaussian random variables with zero mean and unit variance and the mutual information I(ξ;η) in (1) has the form

(2) $$\begin{array}{cc} I(\xi ;\eta ) & =\frac{1}{2}\sum_{i=1}^{r}\log\left(\frac{1}{1-\varrho_{i}^{2}}\right). \end{array}$$

Moreover, $\varrho_{1}\geq \varrho_{2}\geq \ldots \geq \varrho_{r}>0$ denote the positive canonical correlations of ξ and η in descending order, which are obtained by a linear method called canonical correlation analysis that yields the maximum correlations between two sets of random variables (see Section 3). The rank *r* of the cross-covariance matrix $R_{\xi \eta }$ satisfies 0≤r≤min{p,q}, and for r=0 we have i(ξ;η)≡0 almost surely and I(ξ;η)=0. This corresponds to $P_{\xi \eta }=P_{\xi }⊗P_{\eta }$ and the independence of ξ and η such that the resulting information density is deterministic. Throughout the rest of the paper, we exclude this degenerated case when the information density is considered and assume subsequently the setting and notation introduced above with r≥1. As customary notation, we further write R, $N_{0}$, and N to denote the set of real numbers, non-negative integers, and positive integers.

**Main contributions.** Based on (1), we derive in Section 4 series representations of the probability density function (PDF) and the cumulative distribution function (CDF) as well as explicit general formulas for the central moments of the information density i(ξ;η) given subsequently in Theorems 1 to 3. The series representations are useful as they allow tight approximations with errors as low as desired by finite sums as shown in Section 5.2. Moreover, we derive recurrence formulas in Section 5.1 that allow efficient numerical calculations of the series representations in Theorems 1 and 2.

**Theorem** **1** (PDF of information density)**.** *The PDF $f_{i(\xi ;\eta )}$ of the information density i(ξ;η) is given by*

(3) $$\begin{array}{c} f_{i(\xi ;\eta )}\left(x\right)\;=\;\frac{1}{\varrho_{r}\sqrt{\pi }}\sum_{k_{1}=0}^{\infty }\sum_{k_{2}=0}^{\infty }\cdots \sum_{k_{r-1}=0}^{\infty }\left[\prod_{i=1}^{r-1}\frac{\varrho_{r}}{\varrho_{i}}\frac{(2k_{i})!}{{\left(k_{i}!\right)}^{2}4^{k_{i}}}{\left(1-\frac{\varrho_{r}^{2}}{\varrho_{i}^{2}}\right)}^{k_{i}}\right]\times \\ \;\frac{K_{\frac{r-1}{2}+k_{1}+k_{2}+\cdots +k_{r-1}}\left(\frac{\left|x-I(\xi ;\eta )\right|}{\varrho_{r}}\right)}{\Gamma \left(\frac{r}{2}+k_{1}+k_{2}+\cdots +k_{r-1}\right)}{\left(\frac{\left|x-I(\xi ;\eta )\right|}{2\varrho_{r}}\right)}^{\left(\frac{r-1}{2}+k_{1}+k_{2}+\cdots +k_{r-1}\right)},\;x\in R\backslash \left\{I\left(\xi ;\eta \right)\right\}, \end{array}$$

*where Γ(·) denotes the gamma function [9], Sec. 5.2.1 and $K_{\alpha }\left(\cdot \right)$ denotes the modified Bessel function of second kind and order α [9], Sec. 10.25(ii). If r≥2, then $f_{i(\xi ;\eta )}\left(x\right)$ is also well defined for x=I(ξ;η).*

**Theorem** **2** (CDF of information density)**.** *The CDF $F_{i(\xi ;\eta )}$ of the information density i(ξ;η) is given by*

$$F_{i(\xi ;\eta )}\left(x\right)=\left\{\begin{array}{cc} \;\frac{1}{2}-V\left(I(\xi ;\eta )-x\right) & if\;x\leq I(\xi ;\eta )\\ \;\frac{1}{2}+V\left(x-I(\xi ;\eta )\right) & if\;x>I(\xi ;\eta ) \end{array}\right.,$$

*with V(z) defined by*

(4) $$\begin{array}{cc} V(z)= & \sum_{k_{1}=0}^{\infty }\sum_{k_{2}=0}^{\infty }\cdots \sum_{k_{r-1}=0}^{\infty }\left[\prod_{i=1}^{r-1}\frac{\varrho_{r}}{\varrho_{i}}\frac{(2k_{i})!}{{\left(k_{i}!\right)}^{2}4^{k_{i}}}{\left(1-\frac{\varrho_{r}^{2}}{\varrho_{i}^{2}}\right)}^{k_{i}}\right]\frac{z}{2\varrho_{r}}\times \\ \; & [K_{\frac{r-1}{2}+k_{1}+k_{2}+\cdots +k_{r-1}}\left(\frac{z}{\varrho_{r}}\right)L_{\frac{r-3}{2}+k_{1}+k_{2}+\cdots +k_{r-1}}\left(\frac{z}{\varrho_{r}}\right)\\ \; & \;\;+K_{\frac{r-3}{2}+k_{1}+k_{2}+\cdots +k_{r-1}}\left(\frac{z}{\varrho_{r}}\right)L_{\frac{r-1}{2}+k_{1}+k_{2}+\cdots +k_{r-1}}\left(\frac{z}{\varrho_{r}}\right)],\;z\geq 0, \end{array}$$

*where $L_{\alpha }\left(\cdot \right)$ denotes the modified Struve L function of order α [9], Sec. 11.2.*

The method to obtain the result in Theorem 1 is adopted from Mathai [10], where a series representation of the PDF of the sum of independent gamma distributed random variables is derived. Previous work of Grad and Solomon [11] and Kotz et al. [12] goes in a similar direction as Mathai [10]; however, it is not directly applicable since only the restriction to positive series coefficients is considered there. Using Theorem 1, the series representation of the CDF of the information density in Theorem 2 is obtained. The details of the derivations of Theorems 1 and 2 are provided in Section 4.

**Theorem** **3** (Central moments of information density)**.** *The m-th central moment $E({\left[i\left(\xi ;\eta \right)-I\left(\xi ;\eta \right)\right]}^{m})$ of the information density i(ξ;η) is given by*

$$\begin{array}{cc} E([i(\xi ;\eta ) & {-I\left(\xi ;\eta \right)]}^{m}) \end{array}$$

(5) $$=\left\{\begin{array}{cc} \sum_{\left(m_{1},m_{2},\cdots ,m_{r}\right)\in K_{m,r}^{\left[2\right]}}m!\;\prod_{i=1}^{r}\frac{(2m_{i})!}{4^{m_{i}}{\left(m_{i}!\right)}^{2}}\;\varrho_{i}^{2m_{i}} & \;if\;\;m=2\tilde{m}\\ 0 & \;if\;\;m=2\tilde{m}-1 \end{array}\right.,$$

*for all $\tilde{m}\in N$, where $K_{m,r}^{\left[2\right]}=\left\{\left(m_{1},m_{2},\cdots ,m_{r}\right)\in N_{0}^{r}:2m_{1}+2m_{2}+\cdots +2m_{r}=m\right\}$.*

Pinsker [8], Eq. (9.6.17) provided a formula for $\sum_{i=1}^{r}E\left({\left[\frac{\varrho_{i}}{2}\left({\tilde{\xi }}_{i}^{2}-{\tilde{\eta }}_{i}^{2}\right)\right]}^{m}\right)$, which he called “derived *m*-th central moment” of the information density, where ${\tilde{\xi }}_{i}$ and ${\tilde{\eta }}_{i}$ are given as in (1). These special moments coincide for m=2 with the usual central moments considered in Theorem 3.

The rest of the paper is organized as follows: In Section 2, we discuss important special cases which allow simplified and explicit formulas. In Section 3, we provide some background on the canonical correlation analysis and its application to the calculation of the information density and mutual information for Gaussian random vectors. The proofs of the main Theorems 1 to 3 are given in Section 4. Recurrence formulas, finite sum approximations, and uniform bounds of the approximation error are derived in Section 5, which allow efficient and accurate numerical calculations of the PDF and CDF of the information density. Some examples and illustrations are provided in Section 6, where also the (in)validity of Gaussian approximations is discussed. Finally, Section 7 summarizes the paper. Note that a first version of this paper was published on arXiv as preprint [13].

## 2. Special Cases

### 2.1. Equal Canonical Correlations

A simple but important special case for which the series representations in Theorems 1 and 2 simplify to a single summand and the sum of products in Theorem 3 simplifies to a single product is considered in the following corollary.

**Corollary** **1** (PDF, CDF, and central moments of information density for equal canonical correlations)**.** *If all canonical correlations are equal, i.e.,*

$$\varrho_{1}=\varrho_{2}=\ldots =\varrho_{r},$$

*then we have the following simplifications.*

*(i) The PDF $f_{i(\xi ;\eta )}$ of the information density i(ξ;η) simplifies to*

(6) $$f_{i(\xi ;\eta )}\left(x\right)=\frac{1}{\varrho_{r}\sqrt{\pi }\Gamma \left(\frac{r}{2}\right)}K_{\frac{r-1}{2}}\left(\frac{\left|x-I(\xi ;\eta )\right|}{\varrho_{r}}\right){\left(\frac{\left|x-I(\xi ;\eta )\right|}{2\varrho_{r}}\right)}^{\frac{r-1}{2}},\;x\in R\backslash \left\{I\left(\xi ;\eta \right)\right\},$$

*where I(ξ;η) is given by*

$$I\left(\xi ;\eta \right)=-\frac{r}{2}\log\left(1-\varrho_{r}^{2}\right).$$

*If r≥2, then $f_{i(\xi ;\eta )}\left(x\right)$ is also well defined for x=I(ξ;η).*

*(ii) The CDF $F_{i(\xi ;\eta )}$ of the information density i(ξ;η) is given by*

(7) $$F_{i(\xi ;\eta )}\left(x\right)=\left\{\begin{array}{cc} \;\frac{1}{2}-V\left(I(\xi ;\eta )-x\right) & if\;x\leq I(\xi ;\eta )\\ \;\frac{1}{2}+V\left(x-I(\xi ;\eta )\right) & if\;x>I(\xi ;\eta ) \end{array}\right.,$$

*with V(z) defined by*

(8) $$V\left(z\right)=\frac{z}{2\varrho_{r}}\left[K_{\frac{r-1}{2}}\left(\frac{z}{\varrho_{r}}\right)L_{\frac{r-3}{2}}\left(\frac{z}{\varrho_{r}}\right)+K_{\frac{r-3}{2}}\left(\frac{z}{\varrho_{r}}\right)L_{\frac{r-1}{2}}\left(\frac{z}{\varrho_{r}}\right)\right],\;z\geq 0.$$

*(iii) The m-th central moment $E({\left[i\left(\xi ;\eta \right)-I\left(\xi ;\eta \right)\right]}^{m})$ of the information density i(ξ;η) has the form*

(9) $$\begin{array}{cc} E\left({\left[i\left(\xi ;\eta \right)-I\left(\xi ;\eta \right)\right]}^{m}\right)= & \left\{\begin{array}{cc} \frac{m!}{\left(m\;/\;2\right)!}\;\left(\prod_{j=1}^{m\;/\;2}\left(\frac{r}{2}+j-1\right)\right)\varrho_{r}^{m} & \;if\;\;m=2\tilde{m}\\ 0 & \;if\;\;m=2\tilde{m}-1 \end{array},\right. \end{array}$$

*for all $\tilde{m}\in N$.*

Clearly, if all canonical correlations are equal, then the only nonzero term in the series (3) and (4) occur for $k_{1}=k_{2}=\ldots =k_{r-1}=0$. For this single summand, the product in squared brackets in (3) and (4) is equal to 1 by applying $0^{0}=1$, which yields the results of part (i) and (ii) in Corollary 1. Details of the derivation of part (iii) of the corollary are provided in Section 4.

Note, if all canonical correlations are equal, then we can rewrite (1) as follows:

$$\begin{array}{cc} \nu & =\frac{\varrho_{r}}{2}\left(\sum_{i=1}^{r}{\tilde{\xi }}_{i}^{2}-\sum_{i=1}^{r}{\tilde{\eta }}_{i}^{2}\right)+I\left(\xi ;\eta \right). \end{array}$$

This implies that ν coincides with the distribution of the random variable

$$\begin{array}{cc} \nu_{*} & =\frac{\varrho_{r}}{2}\left(\zeta_{1}-\zeta_{2}\right)+I\left(\xi ;\eta \right), \end{array}$$

where $\zeta_{1}$ and $\zeta_{2}$ are i.i.d. $\chi^{2}$-distributed random variables with *r* degrees of freedom. With this representation, we can obtain the expression of the PDF given in (6) also from [14], Sec. 4.A.4.

**Special cases of Corollary 1.** The case when all canonical correlations are equal is important because it occurs in various situations. The subsequent cases follow from the properties of canonical correlations given in Section 3.

(i) Assume that the random variables $\xi_{1},\xi_{2},\ldots ,\xi_{p},\eta_{1},\eta_{2},\ldots ,\eta_{q}$ are pairwise uncorrelated with the exception of the pairs $\left(\xi_{i},\eta_{i}\right),i=1,2,\ldots ,k\leq \min\left\{p,q\right\}$ for which we have $\mathrm{cor}(\xi_{i},\eta_{i})=\rho \neq 0$, where cor(·,·) denotes the Pearson correlation coefficient. Then, r=k and $\varrho_{i}=\left|\rho \right|$ for all i=1,2,…,r. Note, if p=q=k, then for the previous conditions to hold, it is sufficient that the two-dimensional random vectors $\left(\xi_{i},\eta_{i}\right)$ are i.i.d. However, the identical distribution of the $\left(\xi_{i},\eta_{i}\right)$’s is not necessary. In Laneman [15], the distribution of the information density for an additive white Gaussian noise channel with i.i.d. Gaussian input is determined. This is a special case of the case with i.i.d. random vectors $\left(\xi_{i},\eta_{i}\right)$ just mentioned. In Wu and Jindal [16] and in Buckingham and Valenti [17], an approximation of the information density by a Gaussian random variable is considered for the setting in [15]. A special case very similar to that in [15] is also considered in Polyanskiy et al. [6], Sec. III.J. To the best of the authors’ knowledge, explicit formulas for the general case as considered in this paper are not available yet in the literature.

(ii) Assume that the conditions of part (i) are satisfied. Furthermore, assume that $\hat{A}$ is a real nonsingular matrix of dimension p×p and $\hat{B}$ is a real nonsingular matrix of dimension q×q. Then, the random vectors

$$\begin{array}{c} \hat{\xi }=\hat{A}\;\xi \;\mathrm{and}\;\;\hat{\eta }=\hat{B}\;\eta \end{array}$$

have the same canonical correlations as the random vectors ξ and η, i.e., $\varrho_{i}=\left|\rho \right|$ for all i=1,2,…,k≤min{p,q}.

(iii) If r=1, i.e., if the cross-covariance matrix $R_{\xi ,\eta }$ has rank 1, then Corollary 1 obviously applies. Clearly, the most simple special case with r=1 occurs for p=q=1, where $\varrho_{1}=\left|\mathrm{cor}\left(\xi_{1},\eta_{1}\right)\right|$.

As a simple multivariate example, let the covariance matrix of the random vector $\left(\xi_{1},\xi_{2},\ldots ,\xi_{p},\eta_{1},\eta_{2},\ldots ,\eta_{q}\right)$ be given by the Kac-Murdock–Szegö matrix

$$\begin{array}{c} \left(\begin{array}{cc} R_{\xi } & R_{\xi \eta }\\ R_{\xi \eta } & R_{\eta } \end{array}\right)={\left(\rho^{\left|i-j\right|}\right)}_{i,j=1}^{p+q}\; \end{array}$$

which is related to the covariance function of a first-order autoregressive process, where 0<|ρ|<1. Then, $r=\mathrm{rank}(R_{\xi \eta })=1$ and $\varrho_{1}=\left|\rho \right|$.

(iv) As yet another example, assume p=q and $R_{\xi \eta }=\rho R_{\xi }^{1\;/\;2}R_{\eta }^{1\;/\;2}$ for some 0<|ρ|<1. Then, $\varrho_{i}=\left|\rho \right|$ for i=1,2,…,r=q. Here, $A^{1\;/\;2}$ denotes the square root of the real-valued positive semidefinite matrix *A*, i.e., the unique positive semidefinite matrix *B* such that BB=A.

### 2.2. More on Special Cases with Simplified Formulas

Let us further evaluate the formulas given in Corollary 1 and Theorem 3 for some relevant parameter values.

(i) *Single canonical correlation coefficient.* In the most simple case, there is only a single non-zero canonical correlation coefficient, i.e., r=1. (Recall, in the beginning of the paper, we have excluded the degenerated case when all canonical correlations are zero.) Then, the formulas of the PDF and the *m*-th central moment in Corollary 1 simplify to the form

$$f_{i(\xi ;\eta )}\left(x\right)=\frac{1}{\varrho_{1}\pi }K_{0}\left(\frac{\left|x-I(\xi ;\eta \right|)}{\varrho_{1}}\right),\;x\in R\backslash \left\{I\left(\xi ;\eta \right)\right\},$$

and

(10) $$\begin{array}{cc} E\left({\left[i\left(\xi ;\eta \right)-I\left(\xi ;\eta \right)\right]}^{m}\right)= & \left\{\begin{array}{cc} {\left(\frac{m!}{\left(m\;/\;2\right)!}\right)}^{2}{\left(\frac{\varrho_{1}}{2}\right)}^{m} & \;if\;\;m=2\tilde{m}\\ 0 & \;if\;\;m=2\tilde{m}-1 \end{array}\right. \end{array},$$

for all $\tilde{m}\in N$. A formula equivalent to (10) is also provided by Pinsker [8], Lemma 9.6.1 who considered the special case p=q=1, which implies r=1.

(ii) *Second and fourth central moment.* To demonstrate how the general formula given in Theorem 3 is used, we first consider m=2. In this case, the summation indices $m_{1},m_{2},\ldots ,m_{r}$ have to satisfy $m_{i}=1$ for a single i∈{1,2,…,r}, whereas the remaining $m_{i}$’s have to be zero. Thus, (5) evaluates for m=2 to

(11) $$\begin{array}{c} E\left({\left[i\left(\xi ;\eta \right)-I\left(\xi ;\eta \right)\right]}^{2}\right)=\mathrm{var}\left(i(\xi ;\eta )\right)=\sum_{i=1}^{r}\varrho_{i}^{2}. \end{array}$$

As a slightly more complex example, let m=4. In this case, either we have $m_{i}=2$ for a single i∈{1,2,…,r}, whereas the remaining $m_{i}$’s are zero or we have $m_{i_{1}}=m_{i_{2}}=1$ for two $i_{1}\neq i_{2}\in \left\{1,2,\ldots ,r\right\}$, whereas the remaining $m_{i}$’s have to be zero. Thus, (5) evaluates for m=4 to

$$\begin{array}{c} E\left({\left[i\left(\xi ;\eta \right)-I\left(\xi ;\eta \right)\right]}^{4}\right)=9\sum_{i=1}^{r}\varrho_{i}^{4}+6\sum_{i=2}^{r}\sum_{j=1}^{i-1}\varrho_{i}^{2}\varrho_{j}^{2}. \end{array}$$

(iii) *Even number of equal canonical correlations.* As in Corollary 1, assume that all canonical correlations are equal and additionally assume that the number *r* of canonical correlations is even, i.e., $r=2\tilde{r}$ for some $\tilde{r}\in N$. Then, we can use [9], Secs. 10.47.9, 10.49.1, and 10.49.12 to obtain the following relation for the modified Bessel function $K_{\alpha }\left(\cdot \right)$ of a second kind and order α

(12) $$\begin{array}{c} K_{\frac{r-1}{2}}\left(y\right)=\sqrt{\frac{\pi }{2}}\exp\left(-y\right)\sum_{i=0}^{r\;/\;2-1}\frac{\left(r\;/\;2-1+i\right)!}{\left(r\;/\;2-1-i\right)!\;i!\;2^{i}}\;y^{-(i+\frac{1}{2})},\;y\in \left(0,\infty \right). \end{array}$$

Plugging (12) into (6) and rearranging terms yields the following expression for the PDF of the information density:

$$\begin{array}{cc} f_{i(\xi ;\eta )}\left(x\right)=\;\frac{1}{\varrho_{r}2^{r-1}\left(r\;/\;2-1\right)!} & \exp\left(-\frac{\left|x-I(\xi ;\eta )\right|}{\varrho_{r}}\right)\times \\ \; & \sum_{i=0}^{r\;/\;2-1}\frac{\left(2(r\;/\;2-1)-i\right)!\;2^{i}}{\left(r\;/\;2-1-i\right)!\;i!}{\left(\frac{\left|x-I(\xi ;\eta )\right|}{\varrho_{r}}\right)}^{i},\;x\in R. \end{array}$$

By integration, we obtain for the function V(·) in (8) the expression

$$\begin{array}{cc} V\left(z\right)=\frac{1}{2}\;-\;\frac{1}{2^{r-1}\left(r\;/\;2-1\right)!} & \exp\left(-\frac{z}{\varrho_{r}}\right)\times \\ \; & \sum_{i=0}^{r\;/\;2-1}\frac{\left(2(r\;/\;2-1)-i\right)!\;2^{i}}{\left(r\;/\;2-1-i\right)!}\sum_{j=0}^{i}\frac{1}{(i-j)!}{\left(\frac{z}{\varrho_{r}}\right)}^{i-j},\;z\geq 0. \end{array}$$

Note that these special formulas can also be obtained directly from the results given in [14], Sec. 4.A.3.

To illustrate the principal behavior of the PDF and CDF of the information density for equal canonical correlations, it is instructive to consider the specific value r=2 in the above formulas, which yields

$$\begin{array}{cccc} f_{i(\xi ;\eta )}\left(x\right) & =\frac{1}{2\varrho_{r}}\exp\left(-\frac{\left|x-I(\xi ;\eta )\right|}{\varrho_{r}}\right),\; & & x\in R,\\ V(z) & =\frac{1}{2}\left(1-\exp\left(-\frac{z}{\varrho_{r}}\right)\right),\; & & z\geq 0 \end{array}$$

and r=4, for which we obtain

$$\begin{array}{cccc} f_{i(\xi ;\eta )}\left(x\right) & =\frac{1}{4\varrho_{r}}\exp\left(-\frac{\left|x-I(\xi ;\eta )\right|}{\varrho_{r}}\right)\left(1+\frac{\left|x-I(\xi ;\eta )\right|}{\varrho_{r}}\right),\; & & x\in R,\\ V(z) & =\frac{1}{2}\left(1-\exp\left(-\frac{z}{\varrho_{r}}\right)\left(1+\frac{z}{2\varrho_{r}}\right)\right),\; & & z\geq 0. \end{array}$$

## 3. Mutual Information and Information Density in Terms of Canonical Correlations

First introduced by Hotelling [18], the canonical correlation analysis is a widely used linear method in multivariate statistics to determine the maximum correlations between two sets of random variables. It allows a particularly simple and useful representation of the mutual information and the information density of Gaussian random vectors in terms of the so-called canonical correlations. This representation was first obtained by Gelfand and Yaglom [19] and further extended by Pinsker [8], Ch. 9. For the convenience of the reader, we summarize in this section the essence of the canonical correlation analysis and demonstrate how it is applied to derive the representations in (1) and (2).

The formulation of the canonical correlation analysis given below is particularly suitable for implementations. The corresponding results are given without proof. Details and thorough discussions can be found, e.g., in Härdle and Simar [20], Koch [21], or Timm [22].

Based on the nonsingular covariance matrices $R_{\xi }$ and $R_{\eta }$ of the random vectors $\xi =(\xi_{1},\xi_{2},\ldots ,\xi_{p})$ and $\eta =(\eta_{1},\eta_{2},\ldots ,\eta_{q})$, and the cross-covariance matrix $R_{\xi \eta }$ with rank $r=\mathrm{rank}(R_{\xi \eta })$ satisfying 0≤r≤min{p,q}, define the matrix

$$M=R_{\xi }^{-\frac{1}{2}}R_{\xi \eta }R_{\eta }^{-\frac{1}{2}},$$

where the inverse matrices $R_{\xi }^{-1\;/\;2}={\left(R_{\xi }^{1\;/\;2}\right)}^{-1}$ and $R_{\eta }^{-1\;/\;2}={\left(R_{\eta }^{1\;/\;2}\right)}^{-1}$ can be obtained from diagonalizing $R_{\xi }$ and $R_{\eta }$. Then, the matrix *M* has a singular value decomposition

$$M=UDV^{T},$$

where $V^{T}$ denotes the transpose of *V*. The only non-zero entries $d_{1,1},d_{2,2},\ldots ,d_{r,r}>0$ of the matrix $D={\left(d_{i,j}\right)}_{i,j=1}^{p,q}$ are called canonical correlations of ξ and η, denoted by $\varrho_{i}=d_{i,i},i=1,2,\ldots ,r$. The singular value decomposition can be chosen such that $\varrho_{1}\geq \varrho_{2}\geq \ldots \geq \varrho_{r}$ holds, which is assumed throughout the paper.

Define the random vectors

$$\begin{array}{c} \hat{\xi }=\left({\hat{\xi }}_{1},{\hat{\xi }}_{2},\ldots ,{\hat{\xi }}_{p}\right)=A\;\xi \;\mathrm{and}\;\;\hat{\eta }=\left({\hat{\eta }}_{1},{\hat{\eta }}_{2},\ldots ,{\hat{\eta }}_{q}\right)=B\;\eta , \end{array}$$

where the nonsingular matrices *A* and *B* are given by

$$\begin{array}{c} A=U^{T}R_{\xi }^{-\frac{1}{2}}\;\;\mathrm{and}\;\;B=V^{T}R_{\eta }^{-\frac{1}{2}}. \end{array}$$

Then, the random variables ${\hat{\xi }}_{1},{\hat{\xi }}_{2},\ldots ,{\hat{\xi }}_{p},{\hat{\eta }}_{1},{\hat{\eta }}_{2},\ldots ,{\hat{\eta }}_{q}$ have unit variance and they are pairwise uncorrelated with the exception of the pairs $({\hat{\xi }}_{i},{\hat{\eta }}_{i}),i=1,2,\ldots ,r$ for which we have $\mathrm{cor}\left({\hat{\xi }}_{i},{\hat{\eta }}_{i}\right)=\varrho_{i}$.

Using these results, we obtain for the mutual information and the information density

(13) $$\begin{array}{cccccc} I(\xi ;\eta ) & =I(A\xi ;B\eta ) & & =I(\hat{\xi };\hat{\eta }) & & =\sum_{i=1}^{r}I\left({\hat{\xi }}_{i};{\hat{\eta }}_{i}\right) \end{array}$$

(14) $$\begin{array}{cccccc} i(\xi ;\eta ) & =i(A\xi ;B\eta ) & & =i(\hat{\xi };\hat{\eta }) & & =\sum_{i=1}^{r}i\left({\hat{\xi }}_{i};{\hat{\eta }}_{i}\right)\;(\mathrm{P-almost}\;\mathrm{surely}). \end{array}$$

The first equality in (13) and (14) holds because *A* and *B* are nonsingular matrices, which follows, e.g., from Pinsker [8], Th. 3.7.1. Since we consider the case where ξ and η are jointly Gaussian, $\hat{\xi }$ and $\hat{\eta }$ are jointly Gaussian as well. Therefore, the correlation properties of $\hat{\xi }$ and $\hat{\eta }$ imply that all random variables ${\hat{\xi }}_{i},{\hat{\eta }}_{j}$ are independent except for the pairs $\left({\hat{\xi }}_{i},{\hat{\eta }}_{i}\right)$, i=1,2,…,r. This implies the last equality in (13) and (14), where $i\left({\hat{\xi }}_{1};{\hat{\eta }}_{1}\right),i\left({\hat{\xi }}_{2};{\hat{\eta }}_{2}\right),\ldots ,i\left({\hat{\xi }}_{r};{\hat{\eta }}_{r}\right)$ are independent. The sum representations follow from the chain rules of mutual information and information density and the equivalence between independence and vanishing mutual information and information density.

Since ${\hat{\xi }}_{i}$ and ${\hat{\eta }}_{i}$ are jointly Gaussian with correlation $\mathrm{cor}\left({\hat{\xi }}_{i},{\hat{\eta }}_{i}\right)=\varrho_{i}$, we obtain from (13) and the formula of mutual information for the bivariate Gaussian case the identity (2). Additionally, with ${\hat{\xi }}_{i}$ and ${\hat{\eta }}_{i}$ having zero mean and unit variance, the information density $i({\hat{\xi }}_{i};{\hat{\eta }}_{i})$ is further given by

(15) $$\begin{array}{c} i\left({\hat{\xi }}_{i};{\hat{\eta }}_{i}\right)=-\frac{1}{2}\log\left(1-\varrho_{i}^{2}\right)-\frac{\varrho_{i}^{2}}{2(1-\varrho_{i}^{2})}\left({\hat{\xi }}_{i}^{2}-\frac{2\;{\hat{\xi }}_{i}{\hat{\eta }}_{i}}{\varrho_{i}}+{\hat{\eta }}_{i}^{2}\right),\;i=1,2,\ldots ,r. \end{array}$$

Now assume ${\tilde{\xi }}_{1},{\tilde{\xi }}_{2},\ldots ,{\tilde{\xi }}_{r},{\tilde{\eta }}_{1},{\tilde{\eta }}_{2},$…$,{\tilde{\eta }}_{r}$ are i.i.d. Gaussian random variables with zero mean and unit variance. Then, the distribution of the random vector

$$\begin{array}{c} \frac{1}{\sqrt{2}}\left(\sqrt{1+\varrho_{i}}\;{\tilde{\xi }}_{i}+\sqrt{1-\varrho_{i}}\;{\tilde{\eta }}_{i},\sqrt{1+\varrho_{i}}\;{\tilde{\xi }}_{i}-\sqrt{1-\varrho_{i}}\;{\tilde{\eta }}_{i}\right) \end{array}$$

coincides with the distribution of the random vector $\left({\hat{\xi }}_{i},{\hat{\eta }}_{i}\right)$ for all i=1,2,…,r. Plugging this into (15), we obtain together with (14) that the distribution of the information density i(ξ;η) coincides with the distribution of (1).

## 4. Proof of Main Results

### 4.1. Auxiliary Results

To prove Theorem 1, the following lemma regarding the characteristic function of the information density is utilized. The results of the lemma are also used in Ibragimov and Rozanov [23] but without proof. Therefore, the proof is given below for completeness.

**Lemma 1**  (Characteristic function of (shifted) information density)**.** *The characteristic function of the shifted information density i(ξ;η)−I(ξ;η) is equal to the characteristic function of the random variable*

(16) $$\begin{array}{cc} \tilde{\nu } & =\frac{1}{2}\sum_{i=1}^{r}\varrho_{i}\left({\tilde{\xi }}_{i}^{2}-{\tilde{\eta }}_{i}^{2}\right), \end{array}$$

*where ${\tilde{\xi }}_{1},{\tilde{\xi }}_{2},\ldots ,{\tilde{\xi }}_{r},{\tilde{\eta }}_{1},{\tilde{\eta }}_{2},$…$,{\tilde{\eta }}_{r}$ are i.i.d. Gaussian random variables with zero mean and unit variance, and $\varrho_{1},\varrho_{2},\ldots ,\varrho_{r}$ are the canonical correlations of ξ and η. The characteristic function of $\tilde{\nu }$ is given by*

(17) $$\varphi_{\tilde{\nu }}\left(t\right)=\prod_{i=1}^{r}\frac{1}{\sqrt{1+\varrho_{i}^{2}t^{2}}},\;t\in R.$$

**Proof.**  Due to (1), the distribution of the shifted information density i(ξ;η)−I(ξ;η) coincides with the distribution of the random variable $\tilde{\nu }$ in (16) such that the characteristic functions of i(ξ;η)−I(ξ;η) and $\tilde{\nu }$ are equal.

It is a well known fact that ${\tilde{\xi }}_{i}^{2}$ and ${\tilde{\eta }}_{i}^{2}$ in (16) are chi-squared distributed random variables with one degree of freedom from which we obtain that the weighted random variables $\varrho_{i}{\tilde{\xi }}_{i}^{2}/2$ and $\varrho_{i}{\tilde{\eta }}_{i}^{2}/2$ are gamma distributed with a scale parameter of $1/\varrho_{i}$ and shape parameter of 1/2. The characteristic function of these random variables therefore admits the form

$$\varphi_{\frac{\varrho_{i}}{2}{\tilde{\xi }}_{i}^{2}}\left(t\right)={\left(1-\varrho_{i}jt\right)}^{-\frac{1}{2}}.$$

Further, from the identity $\varphi_{-\varrho_{i}{\tilde{\xi }}_{i}^{2}/2}\left(t\right)=\varphi_{\varrho_{i}{\tilde{\xi }}_{i}^{2}/2}\left(-t\right)$ for the characteristic function and from the independence of ${\tilde{\xi }}_{i}$ and ${\tilde{\eta }}_{i}$, we obtain the characteristic function of ${\tilde{\nu }}_{i}=\varrho_{i}\left({\tilde{\xi }}_{i}^{2}-{\tilde{\eta }}_{i}^{2}\right)/2$ to be given by

$$\varphi_{{\tilde{\nu }}_{i}}\left(t\right)={\left(1-\varrho_{i}jt\right)}^{-\frac{1}{2}}{\left(1+\varrho_{i}jt\right)}^{-\frac{1}{2}}={\left(1+\varrho_{i}^{2}t^{2}\right)}^{-\frac{1}{2}}.$$

Finally, because $\tilde{\nu }$ in (16) is given by the sum of the independent random variables ${\tilde{\nu }}_{i}$, the characteristic function of $\tilde{\nu }$ results from multiplying the individual characteristic functions of the random variables ${\tilde{\nu }}_{i}$. By doing so, we obtain (17). □

As further auxiliary result, the subsequent proposition providing properties of the modified Bessel function $K_{\alpha }$ of second kind and order α will be used to prove the main results.

**Proposition** **1** (Properties related to the function $K_{\alpha }$)**.** *For all α∈R, the function*

$$\begin{array}{c} y\mapsto y^{\alpha }K_{\alpha }\left(y\right),\;y\in \left(0,\infty \right), \end{array}$$

*where $K_{\alpha }\left(\cdot \right)$ denotes the modified Bessel function of second kind and order α [9], Sec. 10.25(ii), is strictly positive and strictly monotonically decreasing. Furthermore, if α>0, then we have*

(18) $$\begin{array}{c} \lim_{y\to +0}y^{\alpha }K_{\alpha }\left(y\right)=\underset{y\in (0,\infty )}{\sup}y^{\alpha }K_{\alpha }\left(y\right)=\Gamma \left(\alpha \right)2^{\alpha -1}. \end{array}$$

**Proof.**  If α∈R is fixed, then $K_{\alpha }\left(y\right)$ is strictly positive and strictly monotonically decreasing w. r. t. y∈(0,∞) due to [9], Secs. 10.27.3 and 10.37. Furthermore, we obtain

$$\begin{array}{c} \frac{\;dy^{\alpha }K_{\alpha }\left(y\right)}{\;dy}=-y^{\alpha }K_{\alpha -1}\left(y\right),\;y\in \left(0,\infty \right) \end{array}$$

by applying the rules to calculate derivatives of Bessel functions given in [9], Sec. 10.29(ii). It follows that $y^{\alpha }K_{\alpha }\left(y\right)$ is strictly positive and strictly monotonically decreasing w. r. t. y∈(0,∞) for all fixed α∈R.

Consider now the Basset integral formula as given in [9], Sec. 10.32.11

(19) $$\begin{array}{c} K_{\alpha }\left(yz\right)=\frac{\Gamma \left(\alpha +\frac{1}{2}\right){\left(2z\right)}^{\alpha }}{y^{\alpha }\sqrt{\pi }}\int_{u=0}^{\infty }\frac{\cos(uy)}{{\left(u^{2}+z^{2}\right)}^{\alpha +\frac{1}{2}}}\;du \end{array}$$

for $|\arg\left(z\right)|<\pi \;/\;2,\;y>0,\;\alpha >-\frac{1}{2}$ and the integral

(20) $$\begin{array}{c} \int_{u=0}^{\infty }\frac{1}{{\left(u^{2}+1\right)}^{\alpha +\frac{1}{2}}}\;du=\frac{\sqrt{\pi }\;\Gamma \left(\alpha \right)}{2\;\Gamma \left(\alpha +\frac{1}{2}\right)} \end{array}$$

for α>0, where the equality holds due to [24], Secs. 3.251.2 and 8.384.1. Using (19) and (20), we obtain

$$\begin{array}{cc} \lim_{y\to +0}y^{\alpha }K_{\alpha }\left(y\right) & =\lim_{y\to +0}\frac{\Gamma \left(\alpha +\frac{1}{2}\right)2^{\alpha }}{\sqrt{\pi }}\int_{u=0}^{\infty }\frac{\cos(uy)}{{\left(u^{2}+1\right)}^{\alpha +\frac{1}{2}}}\;du\\ \; & =\frac{\Gamma \left(\alpha +\frac{1}{2}\right)2^{\alpha }}{\sqrt{\pi }}\int_{u=0}^{\infty }\frac{1}{{\left(u^{2}+1\right)}^{\alpha +\frac{1}{2}}}\;du\;=\;\Gamma \left(\alpha \right)2^{\alpha -1}, \end{array}$$

for all α>0, where we also applied the dominated convergence theorem, which is possible due to $\cos\left(uy\right)/{\left(u^{2}+1\right)}^{\alpha +1\;/\;2}\leq 1/{\left(u^{2}+1\right)}^{\alpha +1\;/\;2}$. Using the previously derived monotonicity, we obtain (18). □

### 4.2. Proof of Theorem 1

To prove Theorem 1, we calculate the PDF $f_{\tilde{\nu }}$ of the random variable $\tilde{\nu }$ introduced in Lemma 1 by inverting the characteristic function $\varphi_{\tilde{\nu }}$ given in (17) via the integral

(21) $$\begin{array}{c} f_{\tilde{\nu }}\left(v\right)=\frac{1}{2\pi }\int_{-\infty }^{\infty }\varphi_{\tilde{\nu }}\left(t\right)\exp\left(-Jtv\right)\;dt,\;v\in R. \end{array}$$

Shifting the PDF of $\tilde{\nu }$ by I(ξ;η), we obtain the PDF $f_{i(\xi ;\eta )}=f_{\tilde{\nu }}\left(x-I\left(\xi ;\eta \right)\right)$, x∈R, of the information density i(ξ;η).

The method used subsequently is based on the work of Mathai [10]. To invert the characteristic function $\varphi_{\tilde{\nu }}$, we expand the factors in (17) as

(22) $$\begin{array}{cc} {\left(1+\varrho_{i}^{2}t^{2}\right)}^{-\frac{1}{2}}\; & ={\left(1+\varrho_{r}^{2}t^{2}\right)}^{-\frac{1}{2}}\frac{\varrho_{r}}{\varrho_{i}}{\left(1+\left(\frac{\varrho_{r}^{2}}{\varrho_{i}^{2}}-1\right){\left(1+\varrho_{r}^{2}t^{2}\right)}^{-1}\right)}^{-\frac{1}{2}} \end{array}$$

(23) =1+ϱr2t2−12∑k=0∞(−1)k−1/2kϱrϱi1−ϱr2ϱi2k1+ϱr2t2−k.

In (23), we have used the binomial series

(24) (1+y)a=∑k=0∞akyk

where a∈R. The series is absolutely convergent for |y|<1 and

(25) ak=∏ℓ=1ka−ℓ+1ℓ,k∈N,

denotes the generalized binomial coefficient with a0=1. Since

(26) $$\begin{array}{c} \left|\left(1-\frac{\varrho_{r}^{2}}{\varrho_{i}^{2}}\right){\left(1+\varrho_{r}^{2}t^{2}\right)}^{-1}\right|<1 \end{array}$$

holds for all t∈R, the series in (23) is absolutely convergent for all t∈R. Using the expansion in (23) and the absolute convergence together with the identity

(27) −1/2k=(−1)k(2k)!(k!)24k

we can rewrite the characteristic function $\varphi_{\tilde{\nu }}$ as

(28) $$\begin{array}{cc} \varphi_{\tilde{\nu }}\left(t\right)=\sum_{k_{1}=0}^{\infty }\sum_{k_{2}=0}^{\infty }\cdots \sum_{k_{r-1}=0}^{\infty } & \left[\prod_{i=1}^{r-1}\frac{\varrho_{r}}{\varrho_{i}}\frac{(2k_{i})!}{{\left(k_{i}!\right)}^{2}4^{k_{i}}}{\left(1-\frac{\varrho_{r}^{2}}{\varrho_{i}^{2}}\right)}^{k_{i}}\right]\times \\ \; & \;{\left(1+\varrho_{r}^{2}t^{2}\right)}^{-\left(\frac{r}{2}+k_{1}+k_{2}+\cdots +k_{r-1}\right)},\;t\in R. \end{array}$$

To obtain the PDF $f_{\tilde{\nu }}$, we evaluate the inversion integral (21) based on the series representation in (28). Since every series in (28) is absolutely convergent, we can exchange summation and integration. Let $\beta =\frac{r}{2}+k_{1}+k_{2}+\cdots +k_{r-1}$. Then, by symmetry, we have for the integral of a summand

(29) $$\int_{t=-\infty }^{\infty }\frac{\exp\left(-Jtv\right)}{{\left(1+\varrho_{r}^{2}t^{2}\right)}^{\beta }}\;dt=2\int_{t=0}^{\infty }\frac{\cos\left(tv\right)}{{\left(1+\varrho_{r}^{2}t^{2}\right)}^{\beta }}\;dt=\frac{2}{\varrho_{r}}\int_{u=0}^{\infty }\frac{\cos\left(uv/\varrho_{r}\right)}{{\left(1+u^{2}\right)}^{\beta }}\;du,$$

where the second equality is a result of the substitution $t=u/\varrho_{r}$. By setting z=1, $\alpha =\beta -\frac{1}{2}\geq 0$ and $y=v/\varrho_{r}$ in the Basset integral formula given in (19) in the proof of Proposition 1 and using the symmetry with respect to *v*, we can evaluate (29) to the following form:

(30) $$\int_{t=-\infty }^{\infty }\frac{\exp\left(-Jtv\right)}{{\left(1+\varrho_{r}^{2}t^{2}\right)}^{\beta }}\;dt=\frac{\sqrt{\pi }}{\Gamma \left(\beta \right)2^{\beta -\frac{3}{2}}\varrho_{r}^{\beta +\frac{1}{2}}}K_{\beta -\frac{1}{2}}\left(\frac{\left|v\right|}{\varrho_{r}}\right){\left|v\right|}^{\beta -\frac{1}{2}},\;v\in R\backslash \left\{0\right\}.$$

Combining (21), (28), and (30) yields

(31) $$\begin{array}{c} f_{\tilde{\nu }}\left(v\right)=\frac{1}{2\sqrt{\pi }}\sum_{k_{1}=0}^{\infty }\sum_{k_{2}=0}^{\infty }\cdots \sum_{k_{r-1}=0}^{\infty }\left[\prod_{i=1}^{r-1}\frac{\varrho_{r}}{\varrho_{i}}\frac{(2k_{i})!}{{\left(k_{i}!\right)}^{2}4^{k_{i}}}{\left(1-\frac{\varrho_{r}^{2}}{\varrho_{i}^{2}}\right)}^{k_{i}}\right]\times \\ \frac{K_{\frac{r-1}{2}+k_{1}+k_{2}+\cdots +k_{r-1}}\left(\frac{\left|v\right|}{\varrho_{r}}\right){\left|v\right|}^{\left(\frac{r-1}{2}+k_{1}+k_{2}+\cdots +k_{r-1}\right)}}{\Gamma \left(\frac{r}{2}+k_{1}+k_{2}+\cdots +k_{r-1}\right)2^{\left(\frac{r-3}{2}+k_{1}+k_{2}+\cdots +k_{r-1}\right)}\varrho_{r}^{\left(\frac{r+1}{2}+k_{1}+k_{2}+\cdots +k_{r-1}\right)}},\;v\in R\backslash \left\{0\right\}. \end{array}$$

Slightly rearranging terms and shifting $f_{\tilde{\nu }}\left(\cdot \right)$ by I(ξ;η) yields (3).

It remains to show that $f_{i(\xi ;\eta )}\left(x\right)$ is also well defined for x=I(ξ;η) if r≥2. Indeed, if r≥2, then we can use Proposition 1 to obtain

$$\begin{array}{c} \lim_{x\to I(\xi ;\eta )}f_{i(\xi ;\eta )}\left(x\right)=\frac{1}{2\varrho_{r}\sqrt{\pi }}\sum_{k_{1}=0}^{\infty }\sum_{k_{2}=0}^{\infty }\cdots \sum_{k_{r-1}=0}^{\infty }\left[\prod_{i=1}^{r-1}\frac{\varrho_{r}}{\varrho_{i}}\frac{(2k_{i})!}{{\left(k_{i}!\right)}^{2}4^{k_{i}}}{\left(1-\frac{\varrho_{r}^{2}}{\varrho_{i}^{2}}\right)}^{k_{i}}\right]\times \\ \frac{\Gamma \left(\frac{r-1}{2}+k_{1}+k_{2}+\cdots +k_{r-1}\right)}{\Gamma \left(\frac{r-1}{2}+k_{1}+k_{2}+\cdots +k_{r-1}+\frac{1}{2}\right)} \end{array}$$

where we used the exchangeability of the limit and the summation due to the absolute convergence of the series. Since $\Gamma \left(\alpha \right)/\Gamma \left(\alpha +\frac{1}{2}\right)$ is decreasing w. r. t. $\alpha \geq \frac{1}{2}$, we have

$$\begin{array}{c} \frac{\Gamma \left(\frac{r-1}{2}+k_{1}+k_{2}+\cdots +k_{r-1}\right)}{\Gamma \left(\frac{r-1}{2}+k_{1}+k_{2}+\cdots +k_{r-1}+\frac{1}{2}\right)}\leq \frac{\Gamma \left(\frac{r-1}{2}\right)}{\Gamma \left(\frac{r-1}{2}+\frac{1}{2}\right)}\leq \sqrt{\pi }. \end{array}$$

Then, with (69) in the proof of Theorem 4, it follows that $\lim_{x\to I(\xi ;\eta )}f_{i(\xi ;\eta )}\left(x\right)$ exists and is finite. □

### 4.3. Proof of Theorem 2

To prove Theorem 2, we calculate the CDF $F_{\tilde{\nu }}$ of the random variable $\tilde{\nu }$ introduced in Lemma 1 by integrating the PDF $f_{\tilde{\nu }}$ given in (31). Shifting the CDF of $\tilde{\nu }$ by I(ξ;η), we obtain the CDF $F_{i(\xi ;\eta )}\left(x\right)=F_{\tilde{\nu }}\left(x-I\left(\xi ;\eta \right)\right),x\in R$, of the information density i(ξ;η). Using the symmetry of $f_{\tilde{\nu }}$, we can write

$$F_{\tilde{\nu }}\left(z\right)=P\left(\tilde{\nu }\leq z\right)=\left\{\begin{array}{cc} \frac{1}{2}-\int_{v=0}^{-z}f_{\tilde{\nu }}\left(v\right)\;dv & \mathrm{for}\;z\leq 0\\ \frac{1}{2}+\int_{v=0}^{z}f_{\tilde{\nu }}\left(v\right)\;dv & \mathrm{for}\;z>0 \end{array}\right..$$

It is therefore sufficient to evaluate the integral

(32) $$\begin{array}{c} V\left(z\right):=\int_{v=0}^{z}f_{\tilde{\nu }}\left(v\right)\;dv \end{array}$$

for z≥0. To calculate the integral (32), we plug (31) into (32) and exchange integration and summation, which is justified by the monotone convergence theorem. To evaluate the integral of a summand, consider the following identity

(33) $$\begin{array}{c} \int_{x=0}^{z}x^{\alpha }K_{\alpha }\left(x\right)\;dx=2^{\alpha -1}\sqrt{\pi }\Gamma \left(\alpha +\frac{1}{2}\right)z\left[K_{\alpha }\left(z\right)L_{\alpha -1}\left(z\right)+K_{\alpha -1}\left(z\right)L_{\alpha }\left(z\right)\right] \end{array}$$

for α>−1/2 given in [25], Sec. 1.12.1.3, where $L_{\alpha }\left(\cdot \right)$ denotes the modified Struve L function of order α [9], Sec. 11.2. Using (33) with $\alpha =\frac{r-1}{2}+k_{1}+k_{2}+\cdots +k_{r-1}\geq 0$, we obtain (4). □

### 4.4. Proof of Theorem 3

Using the random variable

$$\begin{array}{c} \tilde{\nu }=\sum_{i=1}^{r}{\tilde{\nu }}_{i}\;\mathrm{with}\;{\tilde{\nu }}_{i}=\frac{\varrho_{i}}{2}\left({\tilde{\xi }}_{i}-{\tilde{\eta }}_{i}\right) \end{array}$$

introduced in Lemma 1 and the well-known multinomial theorem [9], Sec. 26.4.9

$$\begin{array}{c} {\left(y_{1}+y_{2}+\ldots y_{r}\right)}^{m}=\sum_{\left(\ell_{1},\ell_{2},\ldots ,\ell_{r}\right)\in K_{m,r}}m!\;\prod_{i=1}^{r}\frac{y_{i}^{\ell_{i}}}{\ell_{i}!}, \end{array}$$

where $K_{m,r}=\left\{\left(\ell_{1},\ell_{2},\cdots ,\ell_{r}\right)\in N_{0}^{r}:\ell_{1}+\ell_{2}+\cdots +\ell_{r}=m\right\}$, we can write the *m*-th central moment of the information density i(ξ;η) as

(34) $$\begin{array}{cc} E({\left[i\left(\xi ;\eta \right)-I\left(\xi ;\eta \right)\right]}^{m}) & =E({\left[\sum_{i=1}^{r}{\tilde{\nu }}_{i}\right]}^{m})\\ \; & =\sum_{\left(\ell_{1},\ell_{2},\ldots ,\ell_{r}\right)\in K_{m,r}}m!\;\prod_{i=1}^{r}\frac{E({\tilde{\nu }}_{i}^{\ell_{i}})}{\ell_{i}!}. \end{array}$$

To obtain the second equality in (34), we have exchanged expectation and summation and additionally used the identity $E\left(\prod_{i=1}^{r}{\tilde{\nu }}_{i}^{\ell_{i}}\right)=\prod_{i=1}^{r}E\left({\tilde{\nu }}_{i}^{\ell_{i}}\right)$, which holds due to the independence of the random variables ${\tilde{\nu }}_{1},{\tilde{\nu }}_{2},\ldots ,{\tilde{\nu }}_{r}$.

Based on the relation between the *ℓ*-th central moment of a random variable and the *ℓ*-th derivative of its characteristic function at 0, we further have

(35) $$\begin{array}{c} E\left({\tilde{\nu }}_{i}^{\ell_{i}}\right)={\left(-j\right)}^{\ell_{i}}\frac{d^{\ell_{i}}}{dt^{\ell_{i}}}\varphi_{{\tilde{\nu }}_{i}}\left(t\right){|}_{t=0}, \end{array}$$

where $\varphi_{{\tilde{\nu }}_{i}}\left(t\right)={\left(1+\varrho_{i}^{2}t^{2}\right)}^{-1\;/\;2},t\in R,$ is the characteristic function of the random variable ${\tilde{\nu }}_{i}$ derived in the proof of Lemma 1. As in the proof of Theorem 1, consider now the binomial series expansion using (24)

φν˜i(t)=1+ϱi2t2−12=∑mi=0∞−1/2miϱit2mi.

The series is absolutely convergent for all $t<\varrho_{i}^{-1}$. Furthermore, consider the Taylor series expansion of the characteristic function $\varphi_{{\tilde{\nu }}_{i}}$ at the point 0

$$\begin{array}{c} \varphi_{{\tilde{\nu }}_{i}}\left(t\right)=\sum_{\ell_{i}=0}^{\infty }\left(\frac{d^{\ell_{i}}}{dt^{\ell_{i}}}\varphi_{{\tilde{\nu }}_{i}}\left(t\right){|}_{t=0}\right)\frac{t^{\ell_{i}}}{\ell_{i}!}. \end{array}$$

Both series expansions must be identical in an open interval around 0 such that we obtain by comparing the series coefficients

dℓidtℓiφν˜i(t)|t=0=ℓi!−1/2ℓi/2ϱiℓiifℓi=2mi0ifℓi=2mi−1

for all $m_{i}\in N$. With this result, (35) evaluates to

(36) $$\begin{array}{cc} E\left({\tilde{\nu }}_{i}^{\ell_{i}}\right)= & \left\{\begin{array}{cc} \frac{{\left(\ell_{i}!\right)}^{2}}{{\left((\ell_{i}\;/\;2)!\right)}^{2}\;4^{\frac{\ell_{i}}{2}}}\varrho_{i}^{\ell_{i}}\; & \;\mathrm{if}\;\ell_{i}=2m_{i}\\ 0 & \;\mathrm{if}\;\ell_{i}=2m_{i}-1 \end{array}\right. \end{array}$$

for all $m_{i}\in N$, where we have additionally used the identity (27).

From (34) and (36) we now obtain $E({\left[i\left(\xi ;\eta \right)-I\left(\xi ;\eta \right)\right]}^{m})=0$ for all $m=2\tilde{m}-1$ with $\tilde{m}\in N$ because, if *m* is odd, then for all $\left(\ell_{1},\ell_{2},\cdots ,\ell_{r}\right)\in K_{m,r}$ at least one of the $\ell_{i}$’s has to be odd. If $m=2\tilde{m}$ with $\tilde{m}\in N$, we obtain from (34) and (36)

$$\begin{array}{cc} \;E({\left[i\left(\xi ;\eta \right)-I\left(\xi ;\eta \right)\right]}^{m})\; & =\sum_{\left(\ell_{1},\ell_{2},\ldots ,\ell_{r}\right)\in K_{m,r}}m!\;\prod_{i=1}^{r}\frac{1}{\ell_{i}!}\frac{{\left(\ell_{i}!\right)}^{2}}{{\left((\ell_{i}\;/\;2)!\right)}^{2}\;4^{\frac{\ell_{i}}{2}}}\varrho_{i}^{\ell_{i}}\\ \; & =\sum_{\left(m_{1},m_{2},\ldots ,m_{r}\right)\in K_{m,r}^{\left[2\right]}}m!\;\prod_{i=1}^{r}\frac{(2m_{i})!}{{\left(m_{i}!\right)}^{2}\;4^{m_{i}}}\varrho_{i}^{2m_{i}}. \end{array}$$

 □

### 4.5. Proof of Part (iii) of Corollary 1

Using the random variable $\tilde{\nu }$ as in the proof of Theorem 3, we can write the *m*-th central moment of the information density i(ξ;η) as

$$\begin{array}{c} E\left({\left[i\left(\xi ;\eta \right)-I\left(\xi ;\eta \right)\right]}^{m}\right)=E\left({\tilde{\nu }}^{m}\right)={\left(-j\right)}^{m}\frac{d^{m}}{dt^{m}}\varphi_{\tilde{\nu }}\left(t\right){|}_{t=0}, \end{array}$$

where the characteristic function $\varphi_{\tilde{\nu }}$ of $\tilde{\nu }$ is given by $\varphi_{\tilde{\nu }}\left(t\right)={\left(1+\varrho_{r}^{2}t^{2}\right)}^{-r\;/\;2},t\in R$, due to Lemma 1 and the equality of all canonical correlations. Using the binomial series and the Taylor series expansion as in the proof of Theorem 3, we obtain

dmdtmφν˜(t)|t=0=m!−r/2m/2ϱrmifm=2m˜0ifm=2m˜−1

for all $\tilde{m}\in N$. Collecting terms and additionally using the definition of the generalized binomial coefficient given in (25) in the proof of Theorem 1 yields (9). □

## 5. Recurrence Formulas and Finite Sum Approximations

If there are at least two distinct canonical correlations, then the PDF $f_{i(\xi ;\eta )}$ and CDF $F_{i(\xi ;\eta )}$ of the information density i(ξ;η) are given by the infinite series in Theorems 1 and 2. If we consider only a finite number of summands in these representations, then we obtain approximations amenable in particular for numerical calculations. However, a direct finite sum approximation of the series in (3) and (4) is rather inefficient since modified Bessel and Struve L functions have to be evaluated for every summand. Therefore, we derive in this section recursive representations, which allow efficient numerical calculations. Furthermore, we derive uniform bounds of the approximation error. Based on the recurrence relations and the error bounds, an implementation in the programming language Python has been developed, which provides an efficient tool to numerically calculate the PDF and CDF of the information density with a predefined accuracy as high as desired. The developed source code as well as illustrating examples are made publicly available in an open access repository on GitLab [26].

Subsequently, we adopt all the previous notation and assume r≥2 and at least two distinct canonical correlations (since otherwise we have the case of Corollary 1, where the series reduce to a single summand).

### 5.1. Recurrence Formulas

The recursive approach developed below is based on the work of Moschopoulos [27], which extended the work of Mathai [10]. First, we rewrite the series representations of the PDF and CDF of the information density given in Theorem 1 and Theorem 2 in a form, which is suitable for recursive calculations. To begin with, we define two functions appearing in the series representations (3) and (4), which involve the modified Bessel function $K_{\alpha }$ of second kind and order α and the modified Struve L function $L_{\alpha }$ of order α. Let us define for all $k\in N_{0}$ the functions $U_{k}$ and $D_{k}$ by

(37) $$\begin{array}{c} U_{k}\left(z\right)=\frac{K_{\frac{r-1}{2}+k}\left(z\right)}{\Gamma \left(\frac{r}{2}+k\right)}{\left(\frac{z}{2}\right)}^{\frac{r-1}{2}+k},\;z\geq 0 \end{array}$$

and

(38) $$\begin{array}{c} D_{k}\left(z\right)=\frac{z}{2\varrho_{r}}\left[K_{\frac{r-1}{2}+k}\left(\frac{z}{\varrho_{r}}\right)L_{\frac{r-3}{2}+k}\left(\frac{z}{\varrho_{r}}\right)+K_{\frac{r-3}{2}+k}\left(\frac{z}{\varrho_{r}}\right)L_{\frac{r-1}{2}+k}\left(\frac{z}{\varrho_{r}}\right)\right],\;z\geq 0. \end{array}$$

Furthermore, we define for all $k\in N_{0}$ the coefficient $\delta_{k}$ by

(39) $$\begin{array}{c} \delta_{k}=\sum_{\left(k_{1},k_{2},\cdots ,k_{r-1}\right)\in K_{k,r-1}}\left[\prod_{i=1}^{r-1}\frac{(2k_{i})!}{{\left(k_{i}!\right)}^{2}4^{k_{i}}}{\left(1-\frac{\varrho_{r}^{2}}{\varrho_{i}^{2}}\right)}^{k_{i}}\right], \end{array}$$

where $K_{k,r-1}=\left\{\left(k_{1},k_{2},\cdots ,k_{r-1}\right)\in N_{0}^{r-1}:k_{1}+k_{2}+\cdots +k_{r-1}=k\right\}$. With these definitions, we obtain the following alternative series representations of (3) and (4) by observing that the multiple summations over the indices $k_{1},k_{2},\ldots ,k_{r-1}$ can be shortened to one summation over the index $k=k_{1}+k_{2}+\ldots +k_{r-1}$.

**Proposition** **2** (Alternative representation of PDF and CDF of the information density)**.** *The PDF $f_{i(\xi ;\eta )}$ of the information density i(ξ;η) given in Theorem 1 has the alternative series representation*

(40) $$\begin{array}{c} f_{i(\xi ;\eta )}\left(x\right)=\frac{1}{\varrho_{r}\sqrt{\pi }}\left(\prod_{i=1}^{r-1}\frac{\varrho_{r}}{\varrho_{i}}\right)\sum_{k=0}^{\infty }\delta_{k}U_{k}\left(\frac{\left|x-I(\xi ;\eta )\right|}{\varrho_{r}}\right),\;x\in R. \end{array}$$

*The function V(·) specifying the CDF $F_{i(\xi ;\eta )}$ of the information density i(ξ;η) as given in Theorem 2 has the alternative series representation*

(41) $$\begin{array}{c} V\left(z\right)=\left(\prod_{i=1}^{r-1}\frac{\varrho_{r}}{\varrho_{i}}\right)\sum_{k=0}^{\infty }\delta_{k}D_{k}\left(z\right),\;z\geq 0. \end{array}$$

Based on the representations in Proposition 2 and with recursive formulas for $U_{k}\left(\cdot \right)$, $D_{k}\left(\cdot \right)$ and $\delta_{k}$, we are in the position to calculate the PDF and CDF of the information density by a single summation over completely recursively defined terms. In the following, we will derive recurrence relations for $U_{k}\left(\cdot \right)$, $D_{k}\left(\cdot \right)$ and $\delta_{k}$, which allow the desired efficient calculations.

**Lemma** **2** (Recurrence formula of the function $U_{k}$)**.** *If for all $k\in N_{0}$ the function $U_{k}$ is defined by (37), then $U_{k}\left(z\right)$ satisfies for all k≥2 and z≥0 the recurrence formula*

(42) $$\begin{array}{c} U_{k}\left(z\right)=\frac{z^{2}}{\left(r+2k-2)(r+2k-4\right)}\;U_{k-2}\left(z\right)+\frac{r+2k-3}{r+2k-2}\;U_{k-1}\left(z\right). \end{array}$$

**Proof.**  First, assume z=0. Based on Proposition 1, we obtain for all $k\in N_{0}$

(43) $$\begin{array}{c} \lim_{z\to +0}U_{k}\left(z\right)=\frac{\Gamma \left(\frac{r-1}{2}+k\right)}{2\;\Gamma \left(\frac{r}{2}+k\right)}, \end{array}$$

such that $U_{k}\left(0\right)$ is well defined and finite. Using the recurrence relation Γ(y+1)=yΓ(y) for the Gamma function [24], Sec. 8.331.1 we have

$$\begin{array}{c} \frac{\Gamma \left(\frac{r-1}{2}+k\right)}{2\;\Gamma \left(\frac{r}{2}+k\right)}=\frac{\left(\frac{r-1}{2}+k-1\right)}{\left(\frac{r}{2}+k-1\right)}\cdot \frac{\Gamma \left(\frac{r-1}{2}+k-1\right)}{2\;\Gamma \left(\frac{r}{2}+k-1\right)}. \end{array}$$

This shows together with (43) that the recurrence formula (42) holds for $U_{k}\left(0\right)$ and k≥2.

Now, assume z>0 and consider the recurrence formula

(44) $$\begin{array}{c} zK_{\alpha }\left(z\right)=zK_{\alpha -2}\left(z\right)+2\left(\alpha -1\right)K_{\alpha -1}\left(z\right) \end{array}$$

for the modified Bessel function of the second kind and order α [24], Sec. 8.486.10. Plugging (44) into (37) for $\alpha =\frac{r-1}{2}+k$ yields for k≥2

(45) $$\begin{array}{cc} U_{k}\left(z\right)=\frac{K_{\frac{r-1}{2}+k-2}\left(z\right)}{\Gamma \left(\frac{r}{2}+k\right)}\; & {\left(\frac{z}{2}\right)}^{\frac{r-1}{2}+k-2}{\left(\frac{z}{2}\right)}^{2}\\ \; & +\frac{\left(\frac{r-1}{2}+k-1\right)K_{\frac{r-1}{2}+k-1}\left(z\right)}{\Gamma \left(\frac{r}{2}+k\right)}{\left(\frac{z}{2}\right)}^{\frac{r-1}{2}+k-1}. \end{array}$$

Using again the relation Γ(y+1)=yΓ(y), we obtain

$$\begin{array}{cc} \Gamma \left(\frac{r}{2}+k\right) & =\left(\frac{r}{2}+k-1\right)\Gamma \left(\frac{r}{2}+k-1\right)=\left(\frac{r}{2}+k-1\right)\left(\frac{r}{2}+k-2\right)\Gamma \left(\frac{r}{2}+k-2\right), \end{array}$$

which yields together with (45) and (37) the recurrence formula (42) for $U_{k}\left(z\right)$ if z>0 and k≥2. □

**Lemma** **3** (Recurrence formula of the function $D_{k}$)**.** *If, for all $k\in N_{0}$, the function $D_{k}$ is defined by (38), then $D_{k}\left(z\right)$ satisfies for all k≥1 and z≥0 the recurrence formula*

(46) $$\begin{array}{c} D_{k}\left(z\right)=D_{k-1}\left(z\right)-\frac{1}{2\sqrt{\pi }\left(\frac{r}{2}+k-1\right)}\;\left(\frac{z}{\varrho_{r}}\right)U_{k-1}\left(\frac{z}{\varrho_{r}}\right), \end{array}$$

*with $U_{k}\left(\cdot \right)$ as defined in (37).*

**Proof.**  First, assume z=0. We have $D_{k}\left(0\right)=0$ for all $k\in N_{0}$ and from the proof of Lemma 2 we have $U_{k}\left(0\right)=\Gamma \left(\frac{r-1}{2}+k\right)/2\;\Gamma \left(\frac{r}{2}+k\right)$ for all $k\in N_{0}$. Thus, the left-hand side and the right-hand side of (46) are both zero, which shows that (46) holds for z=0 and k≥1.

Now, assume z>0 and consider the recurrence formula

$$\begin{array}{c} zL_{\alpha }\left(z\right)=zL_{\alpha -2}\left(z\right)-2\left(\alpha -1\right)L_{\alpha -1}\left(z\right)-\frac{2^{1-\alpha }z^{\alpha }}{\sqrt{\pi }\Gamma \left(\alpha +\frac{1}{2}\right)} \end{array}$$

for the modified Struve L function of order α [9], Sec. 11.4.25. Together with the recurrence formula (44) for the modified Bessel function of the second kind and order α, we obtain

$$\begin{array}{cc} zL_{\alpha }\left(z\right)K_{\alpha -1}\left(z\right) & =zL_{\alpha -2}\left(z\right)K_{\alpha -1}\left(z\right)-\;2\left(\alpha -1\right)L_{\alpha -1}\left(z\right)K_{\alpha -1}\left(z\right) \end{array}$$

(47) $$\begin{array}{cccc} & & & -\frac{2^{1-\alpha }z^{\alpha }}{\sqrt{\pi }\Gamma \left(\alpha +\frac{1}{2}\right)}K_{\alpha -1}\left(z\right), \end{array}$$

(48) $$\begin{array}{cccc} zK_{\alpha }\left(z\right)L_{\alpha -1}\left(z\right) & =zK_{\alpha -2}\left(z\right)L_{\alpha -1}\left(z\right) & & +2\left(\alpha -1\right)K_{\alpha -1}\left(z\right)L_{\alpha -1}\left(z\right). \end{array}$$

Plugging (47) and (48) into (38) for $\alpha =\frac{r-1}{2}+k$ yields for k≥1

$$\begin{array}{cc} D_{k}\left(z\right)=\frac{z}{2\varrho_{r}}[ & K_{\frac{r-1}{2}+k-1}\left(\frac{z}{\varrho_{r}}\right)L_{\frac{r-3}{2}+k-1}\left(\frac{z}{\varrho_{r}}\right)+K_{\frac{r-3}{2}+k-1}\left(\frac{z}{\varrho_{r}}\right)L_{\frac{r-1}{2}+k-1}\left(\frac{z}{\varrho_{r}}\right)]\\ \; & -\frac{1}{\sqrt{\pi }\;\Gamma \left(\frac{r}{2}+k\right)}{\left(\frac{z}{2\varrho_{r}}\right)}^{\frac{r-1}{2}+k}K_{\frac{r-1}{2}+k-1}\left(\frac{z}{\varrho_{r}}\right). \end{array}$$

Together with (38), the identity $\Gamma \left(\frac{r}{2}+k\right)=\left(\frac{r}{2}+k-1\right)\Gamma \left(\frac{r}{2}+k-1\right)$, and the definition of the function $U_{k}\left(\cdot \right)$ in (37), we obtain the recurrence formula (46) for $D_{k}\left(z\right)$ if z>0 and k≥1. □

**Lemma** **4** (Recursive formula of the coefficient $\delta_{k}$)**.** *The coefficient $\delta_{k}$ defined by (39) satisfies for all $k\in N_{0}$ the recurrence formula*

(49) $$\begin{array}{c} \delta_{k+1}=\frac{1}{k+1}\sum_{j=1}^{k+1}j\;\gamma_{j}\;\delta_{k+1-j}, \end{array}$$

*where $\delta_{0}=1$ and*

(50) $$\begin{array}{c} \gamma_{j}=\sum_{i=1}^{r-1}\frac{1}{2j}{\left(1-\frac{\varrho_{r}^{2}}{\varrho_{i}^{2}}\right)}^{j}. \end{array}$$

For the derivation of Lemma 4, we use an adapted version of the method of Moschopoulos [27] and the following auxiliary result.

**Lemma** **5.** *For $k\in N_{0}$, let g be a real univariate (k+1)-times differentiable function. Then, we have the following recurrence relation for the (k+1)-th derivative of the composite function $h=\exp\left(g\right)$*

(51) h(k+1)=∑j=1k+1kj−1g(j)h(k−j+1),

*where $f^{\left(i\right)}$ denotes the i-th derivative of the function f with $f^{\left(0\right)}=f$.*

**Proof.**  We prove the assertion of Lemma 5 by induction over *k*. First, consider the base case for k=0. In this case, formula (51) gives

$$h^{\left(1\right)}=g^{\left(1\right)}h,$$

which is easily seen to be true.

Assuming formula (51) holds for $h^{\left(k\right)}$, we continue with the case k+1. Application of the product rule leads to

h(k+1)=h(k)(1)=∑j=1kk−1j−1g(j)h(k−j)(1)=∑j=1kk−1j−1g(j+1)h(k−j)+∑j=1kk−1j−1g(j)h(k−j+1).

Substitution of $j^{\prime }=j+1$ in the first term gives

h(k+1)=∑j′=2k+1k−1j′−2g(j′)h(k−j′+1)+∑j=1kk−1j−1g(j)h(k−j+1).

With this representation and the identity,

k−1j−2+k−1j−1=kj−1

We finally have

h(k+1)=g(1)h(k)+∑j=2kk−1j−1+k−1j−2g(j)h(k−j+1)+g(k+1)h=k0g(1)h(k)+∑j=2kkj−1g(j)h(k−j+1)+kkg(k+1)h=∑j=1k+1kj−1g(j)h(k−j+1).

This completes the proof of Lemma 5. □

**Proof of Lemma 4**. To prove the recurrence formula (49), we consider the characteristic function

(52) $$\varphi_{\tilde{\nu }}\left(t\right)=\prod_{i=1}^{r}{\left(1+\varrho_{i}^{2}t^{2}\right)}^{-\frac{1}{2}},\;t\in R$$

of the random variable $\tilde{\nu }$ introduced in Lemma 1. On the one hand, the series representation of $\varphi_{\tilde{\nu }}$ given in (28) in the proof of Theorem 1 can be rewritten as follows using the coefficient $\delta_{k}$ defined in (39):

(53) $$\begin{array}{c} \varphi_{\tilde{\nu }}\left(t\right)={\left(1+\varrho_{r}^{2}t^{2}\right)}^{-\frac{r}{2}}\left(\prod_{i=1}^{r-1}\frac{\varrho_{r}}{\varrho_{i}}\right)\sum_{\ell =0}^{\infty }\delta_{\ell }{\left(1+\varrho_{r}^{2}t^{2}\right)}^{-\ell },\;t\in R. \end{array}$$

On the other hand, recall the expansion of ${\left(1+\varrho_{i}^{2}t^{2}\right)}^{-\frac{1}{2}}$ given in (22), which yields together with (52) and the application of the natural logarithm the identity

(54) $$\begin{array}{cc} \log\left(\varphi_{\tilde{\nu }}\left(t\right)\right)=\; & \log\left({\left(1+\varrho_{r}^{2}t^{2}\right)}^{-\frac{r}{2}}\left(\prod_{i=1}^{r-1}\frac{\varrho_{r}}{\varrho_{i}}\right)\right)\\ \; & +\sum_{i=1}^{r-1}\log\left({\left(1+\left(\frac{\varrho_{r}^{2}}{\varrho_{i}^{2}}-1\right){\left(1+\varrho_{r}^{2}t^{2}\right)}^{-1}\right)}^{-\frac{1}{2}}\right). \end{array}$$

Now consider the power series

(55) $$\log\left(1+y\right)=\sum_{\ell =1}^{\infty }\frac{{\left(-1\right)}^{\ell +1}}{\ell }y^{\ell },$$

which is absolutely convergent for |y|<1. With the same arguments as in the proof of Theorem 1, in particular due to (26), we can apply the series expansion (55) to the second term on the right-hand side of (54) to obtain the absolutely convergent series representation

(56) $$\begin{array}{c} \begin{array}{cc} \log\left(\varphi_{\tilde{\nu }}\left(t\right)\right)= & \log\left({\left(1+\varrho_{r}^{2}t^{2}\right)}^{-\frac{r}{2}}\left(\prod_{i=1}^{r-1}\frac{\varrho_{r}}{\varrho_{i}}\right)\right)+\sum_{\ell =1}^{\infty }\gamma_{\ell }{\left(1+\varrho_{r}^{2}t^{2}\right)}^{-\ell }, \end{array} \end{array}$$

where we have further used the definition of $\gamma_{\ell }$ given in (50). Applying the exponential function to both sides of (56) then yields the following expression for the characteristic function $\varphi_{\tilde{\nu }}$.

(57) $$\begin{array}{cc} \varphi_{\tilde{\nu }}\left(t\right)= & {\left(1+\varrho_{r}^{2}t^{2}\right)}^{-\frac{r}{2}}\left(\prod_{i=1}^{r-1}\frac{\varrho_{r}}{\varrho_{i}}\right)\exp\left(\sum_{\ell =1}^{\infty }\gamma_{\ell }{\left(1+\varrho_{r}^{2}t^{2}\right)}^{-\ell }\right) \end{array}$$

Comparing (53) and (57) yields the identity

(58) $$\sum_{\ell =0}^{\infty }\delta_{\ell }{\left(1+\varrho_{r}^{2}t^{2}\right)}^{-\ell }=\exp\left(\sum_{\ell =1}^{\infty }\gamma_{\ell }{\left(1+\varrho_{r}^{2}t^{2}\right)}^{-\ell }\right).$$

We now define $x={\left(1+\varrho_{r}^{2}t^{2}\right)}^{-1}$ and take the (k+1)-th derivative w. r. t. *x* on both sides of (58) using the identity

(59) $$\frac{d^{m}}{dx^{m}}\left(\sum_{\ell =0}^{\infty }a_{\ell }x^{\ell }\right)=\frac{d^{m}}{dx^{m}}\left(\sum_{\ell =1}^{\infty }a_{\ell }x^{\ell }\right)=\sum_{\ell =m}^{\infty }\frac{\ell !}{(\ell -m)!}a_{\ell }x^{\ell -m}$$

for the *m*-th derivative of a power series $\sum_{\ell =0}^{\infty }a_{\ell }x^{\ell }$. For the left-hand side of (58), we obtain

(60) $$\begin{array}{c} \frac{d^{k+1}}{dx^{k+1}}\left(\sum_{\ell =0}^{\infty }\delta_{\ell }x^{\ell }\right)=\sum_{\ell =k+1}^{\infty }\frac{\ell !}{(\ell -k-1)!}\delta_{\ell }x^{\ell -k-1}. \end{array}$$

For the right-hand side of (58), we obtain

(61) dk+1dxk+1exp∑ℓ=1∞γℓxℓ=∑j=1k+1kj−1djdxj∑ℓ=1∞γℓxℓdk−j+1dxk−j+1exp∑ℓ=1∞γℓxℓ=∑j=1k+1kj−1djdxj∑ℓ=1∞γℓxℓdk−j+1dxk−j+1∑ℓ=0∞δℓxℓ=∑j=1k+1kj−1∑ℓ=j∞ℓ!γℓ(ℓ−j)!xℓ−j×∑ℓ=k+1−j∞ℓ!δℓ(ℓ−k+j−1)!xℓ−k+j−1,

where we used Lemma 5 and the identities (58) and (59). From the equality

$$\begin{array}{c} \frac{d^{k+1}}{dx^{k+1}}\left(\sum_{\ell =0}^{\infty }\delta_{\ell }x^{\ell }\right)=\frac{d^{k+1}}{dx^{k+1}}\left(\exp\left(\sum_{\ell =1}^{\infty }\gamma_{\ell }x^{\ell }\right)\right) \end{array}$$

and the evaluation of the right-hand side of (60) and (61), we obtain

(k+1)!δk+1x0+…x1+…x2…=∑j=1k+1kj−1j!γj(k+1−j)!δk+1−jx0+…x1+…x2…

Comparing the coefficients for $x^{0}$ finally yields

δk+1=1(k+1)!∑j=1k+1kj−1j!γj(k+1−j)!δk+1−j=1(k+1)!∑j=1k+1k!(j−1)!(k+1−j)!j!γj(k+1−j)!δk+1−j=1(k+1)∑j=1k+1jγjδk+1−j.

This completes the proof of Lemma 4. □

### 5.2. Finite Sum Approximations

The results in the previous Section 5.1 can be used in the following way for efficient numerical calculations. Consider

(62) $$\begin{array}{c} {\hat{f}}_{i(\xi ;\eta )}\left(x,n\right)=\frac{1}{\varrho_{r}\sqrt{\pi }}\left(\prod_{i=1}^{r-1}\frac{\varrho_{r}}{\varrho_{i}}\right)\sum_{k=0}^{n}\delta_{k}U_{k}\left(\frac{\left|x-I(\xi ;\eta )\right|}{\varrho_{r}}\right),\;x\in R \end{array}$$

for $n\in N_{0}$, i.e., the finite sum approximation of the PDF given in (40). To calculate ${\hat{f}}_{i(\xi ;\eta )}\left(x,n\right)$, first calculate $U_{0}\left(|x-I\left(\xi ;\eta \right)|/\varrho_{r}\right)$ and $U_{1}\left(|x-I\left(\xi ;\eta \right)|/\varrho_{r}\right)$ using (37). Then, use the recurrence formulas (42) and (49) to calculate the remaining summands in (62). The great advantage of this approach is that only two evaluations of the modified Bessel function are required and for the rest of the calculations efficient recursive formulas are employed making the numerical computations efficient.

Similarly, consider

(63) $$\begin{array}{cc} {\hat{F}}_{i(\xi ;\eta )}\left(x,n\right)= & \left\{\begin{array}{cc} \begin{array}{c} \;\frac{1}{2}-\hat{V}\left(I(\xi ;\eta )-x,n\right) \end{array} & \mathrm{if}\;x\leq I(\xi ;\eta )\\ \;\frac{1}{2}+\hat{V}\left(x-I(\xi ;\eta ),n\right) & \mathrm{if}\;x>I(\xi ;\eta ) \end{array}\right., \end{array}$$

(64) $$\begin{array}{c} \mathrm{with}\;\hat{V}(z,n)=\left(\prod_{i=1}^{r-1}\frac{\varrho_{r}}{\varrho_{i}}\right)\sum_{k=0}^{n}\delta_{k}D_{k}\left(z\right),\;z\geq 0, \end{array}$$

for $n\in N_{0}$, i.e., the finite sum approximation of the alternative representation of the CDF of the information density, where $\hat{V}\left(z,n\right)$ is the finite sum approximation of the function V(·) given in (41). To calculate ${\hat{F}}_{i(\xi ;\eta )}\left(x,n\right)$, first calculate $D_{0}\left(z\right)$, $U_{0}\left(z\;/\;\varrho_{r}\right)$, and $U_{1}\left(z\;/\;\varrho_{r}\right)$ for z=I(ξ;η)−x or z=x−I(ξ;η) using (37) and (38). Then, use the recurrence formulas (42), (46), and (49) to calculate the remaining summands in (64). This approach requires only three evaluations of the modified Bessel and Struve L function resulting in efficient numerical calculations also for the CDF of the information density.

The following theorem provides suitable bounds to evaluate and control the error related to the introduced finite sum approximations.

**Theorem** **4** (Bounds of the approximation error for the alternative representation of PDF and CDF)**.** *For the finite sum approximations in (62)–(64) of the alternative representation of the PDF and CDF of the information density as given in Proposition 2, we have for n∈N summands the error bounds*

(65) $$\begin{array}{c} |f_{i(\xi ;\eta )}\left(x\right)-{\hat{f}}_{i(\xi ;\eta )}\left(x,n\right)|\leq \frac{\Gamma \left(\frac{r-1}{2}+n\right)}{2\varrho_{r}\sqrt{\pi }\;\Gamma \left(\frac{r}{2}+n\right)}\left(1-\left(\prod_{i=1}^{r-1}\frac{\varrho_{r}}{\varrho_{i}}\right)\sum_{k=0}^{n}\delta_{k}\right),\;x\in R \end{array}$$

*and*

(66) $$\begin{array}{c} |V\left(z\right)-\hat{V}\left(z,n\right)|\leq \frac{1}{2}\left(1-\left(\prod_{i=1}^{r-1}\frac{\varrho_{r}}{\varrho_{i}}\right)\sum_{k=0}^{n}\delta_{k}\right),\;z\geq 0. \end{array}$$

**Proof.**  From the special case where all canonical correlations are equal, we can conclude from the CDF given in Corollary 1 that the function

(67) $$\begin{array}{c} z\mapsto z\left[K_{\alpha }\left(z\right)L_{\alpha -1}\left(z\right)+K_{\alpha -1}\left(z\right)L_{\alpha }\left(z\right)\right],\;z\geq 0, \end{array}$$

is monotonically increasing for all α=(j−1)/2, j∈N and that further

(68) $$\begin{array}{c} \lim_{z\to \infty }z\left[K_{\alpha }\left(z\right)L_{\alpha -1}\left(z\right)+K_{\alpha -1}\left(z\right)L_{\alpha }\left(z\right)\right]=1 \end{array}$$

holds. Using (68), we obtain from (4)

$$\begin{array}{c} \lim_{z\to \infty }2V\left(z\right)=\sum_{k_{1}=0}^{\infty }\sum_{k_{2}=0}^{\infty }\cdots \sum_{k_{r-1}=0}^{\infty }\left[\prod_{i=1}^{r-1}\frac{\varrho_{r}}{\varrho_{i}}\frac{(2k_{i})!}{{\left(k_{i}!\right)}^{2}4^{k_{i}}}{\left(1-\frac{\varrho_{r}^{2}}{\varrho_{i}^{2}}\right)}^{k_{i}}\right] \end{array}$$

by exchanging the limit and the summation, which is justified by the monotone convergence theorem. Due to the properties of the CDF, we have $\lim_{z\to \infty }2V\left(z\right)=1$, which implies

(69) $$\begin{array}{c} \left(\prod_{i=1}^{r-1}\frac{\varrho_{r}}{\varrho_{i}}\right)\sum_{k=0}^{\infty }\delta_{k}=\sum_{k_{2}=0}^{\infty }\cdots \sum_{k_{r-1}=0}^{\infty }\left[\prod_{i=1}^{r-1}\frac{\varrho_{r}}{\varrho_{i}}\frac{(2k_{i})!}{{\left(k_{i}!\right)}^{2}4^{k_{i}}}{\left(1-\frac{\varrho_{r}^{2}}{\varrho_{i}^{2}}\right)}^{k_{i}}\right]=1, \end{array}$$

where the first equality follows from the definition of the coefficient $\delta_{k}$ in (39).

We now obtain with (41) and (64)

$$\begin{array}{cc} |V\left(z\right)-\hat{V}\left(z,n\right)|=\;\left(\prod_{i=1}^{r-1}\frac{\varrho_{r}}{\varrho_{i}}\right)\sum_{k=n+1}^{\infty }\delta_{k}D_{k}\left(z\right)\leq \; & \left(\prod_{i=1}^{r-1}\frac{\varrho_{r}}{\varrho_{i}}\right)\sum_{k=n+1}^{\infty }\frac{1}{2}\delta_{k}. \end{array}$$

The inequality follows from the definition of the function $D_{k}\left(\cdot \right)$ in (38), the monotonicity of the function in (67), and from (68). Then, (66) follows from (69).

Similarly, we obtain with (40) and (62)

$$\begin{array}{cc} |f_{i(\xi ;\eta )}\left(x\right)-{\hat{f}}_{i(\xi ;\eta )}\left(x,n\right)|=\;\; & \frac{1}{\varrho_{r}\sqrt{\pi }}\left(\prod_{i=1}^{r-1}\frac{\varrho_{r}}{\varrho_{i}}\right)\sum_{k=n+1}^{\infty }\delta_{k}U_{k}\left(\frac{\left|x-I(\xi ;\eta )\right|}{\varrho_{r}}\right)\\ \leq \;\; & \frac{1}{\varrho_{r}\sqrt{\pi }}\left(\prod_{i=1}^{r-1}\frac{\varrho_{r}}{\varrho_{i}}\right)\sum_{k=n+1}^{\infty }\delta_{k}\frac{\Gamma \left(\frac{r-1}{2}+n\right)}{2\;\Gamma \left(\frac{r}{2}+n\right)}. \end{array}$$

The inequality follows from the definition of the function $U_{k}\left(\cdot \right)$, Proposition 1, and the decreasing monotonicity of $\Gamma \left(\frac{r-1}{2}+k\right)/\Gamma \left(\frac{r}{2}+k\right)$ w. r. t. $k\in N_{0}$. Then, (65) follows from (69). □

**Remark** **1.** *Note that the bound in (65) can be further simplified using the inequality $\Gamma (\alpha )/\Gamma \left(\alpha +1\;/\;2\right)\leq \sqrt{\pi }.$ Further note that the derived error bounds are uniform in the sense that they only depend on the parameters of the given Gaussian distribution and the number of summands considered. As can be seen from (69), the bounds converge to zero as the number of summands jointly increase.*

**Remark** **2** (Relation to Bell polynomials)**.** *Interestingly, the coefficient $\delta_{k}$ can be expressed for all k∈N in the following form*

$$\begin{array}{c} \delta_{k}=\frac{B_{k}\left(\gamma_{1},2\gamma_{2},6\gamma_{3},\ldots ,k!\gamma_{k}\right)}{k!}, \end{array}$$

*where $\gamma_{j}$ is defined in (50), and $B_{k}$ denotes the complete Bell polynomial of order k [28], Sec. 3.3. Even though this is an interesting connection to the Bell polynomials, which provides an explicit formula of $\delta_{k}$, the recursive formula given in Lemma 4 is more efficient for numerical calculations.*

## 6. Numerical Examples and Illustrations

We illustrate the results of this paper with some examples, which all can be verified with the Python implementation publicly available on GITLAB [26].

**Figure 1:**
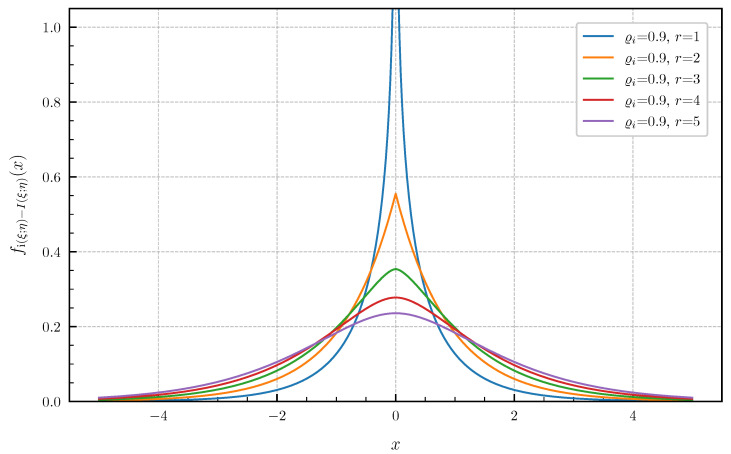
PDF $f_{i(\xi ;\eta )-I(\xi ;\eta )}$ for r∈{1,2,3,4,5} equal canonical correlations $\varrho_{i}=0.9$.

**Figure 2:**
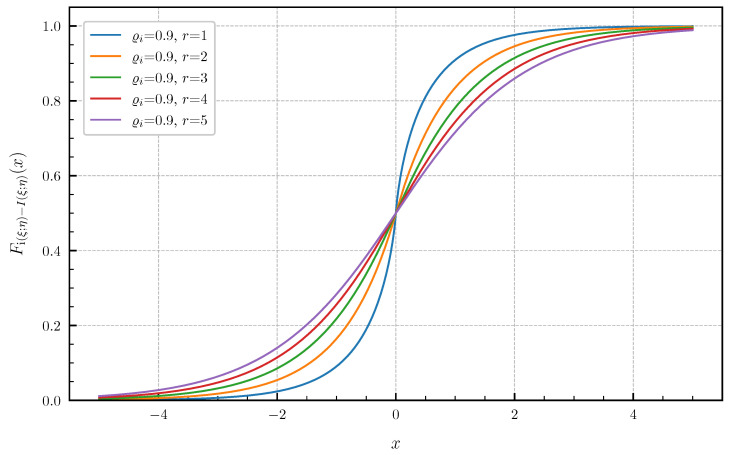
CDF $F_{i(\xi ;\eta )-I(\xi ;\eta )}$ for r∈{1,2,3,4,5} equal canonical correlations $\varrho_{i}=0.9$.

**Figure 3:**
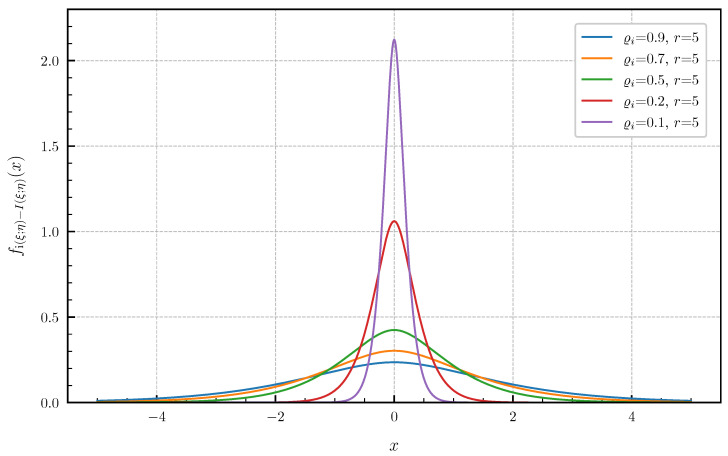
PDF $f_{i(\xi ;\eta )-I(\xi ;\eta )}$ for r=5 equal canonical correlations $\varrho_{i}\in \left\{0.1,0.2,0.5,0.7,0.9\right\}$.

**Figure 4:**
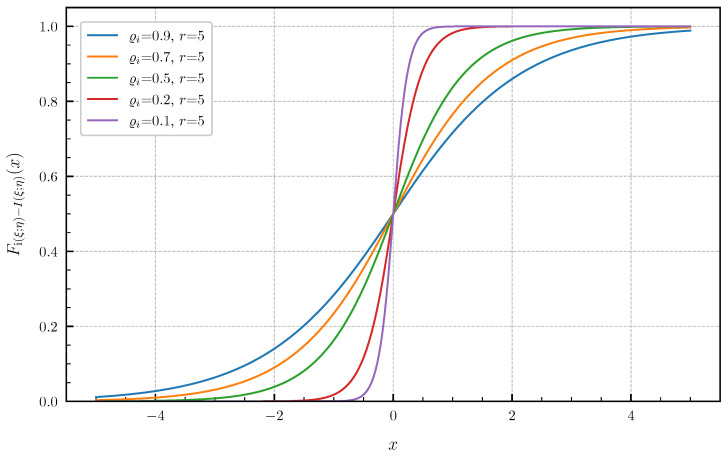
CDF $F_{i(\xi ;\eta )-I(\xi ;\eta )}$ for r=5 equal canonical correlations $\varrho_{i}\in \left\{0.1,0.2,0.5,0.7,0.9\right\}$.

**Figure 5:**
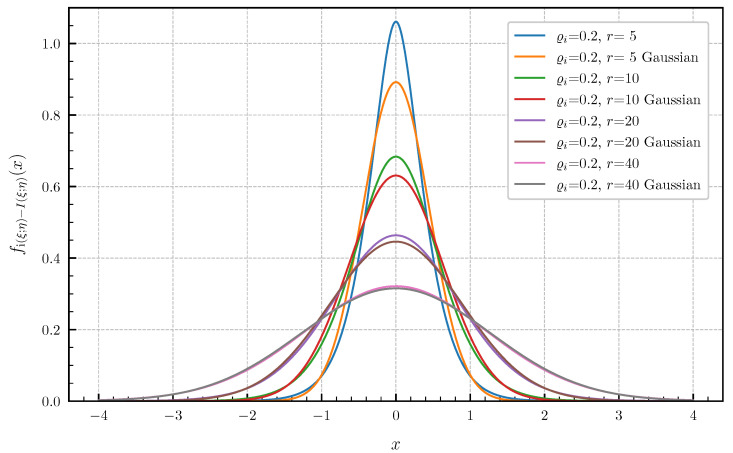
PDF $f_{i(\xi ;\eta )-I(\xi ;\eta )}$ for r∈{5,10,20,40} equal canonical correlations $\varrho_{i}=0.2$ vs. Gaussian approximation.

**Figure 6:**
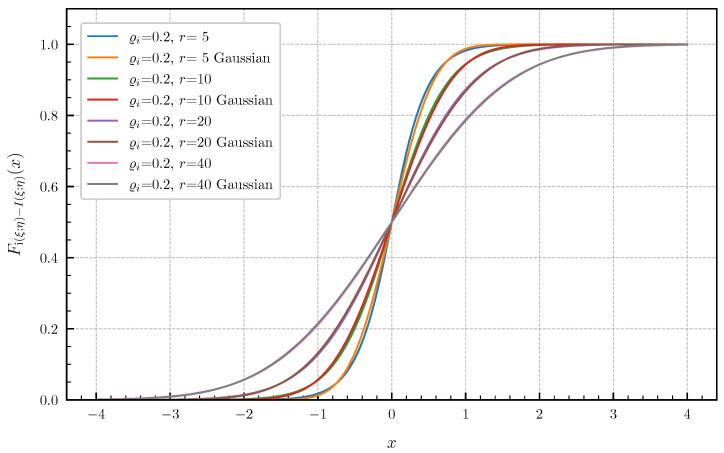
CDF $F_{i(\xi ;\eta )-I(\xi ;\eta )}$ for r∈{5,10,20,40} equal canonical correlations $\varrho_{i}=0.2$ vs. Gaussian approximation.

**Equal canonical correlations.** First, we consider the special case of Corollary 1 when all canonical correlations are equal. The PDF and CDF given by (6) and (7) are illustrated in Figure 1 and Figure 2 in centered form, i.e., shifted by I(ξ;η), for r∈{1,2,3,4,5} and equal canonical correlations $\varrho_{i}=0.9,i=1,\ldots ,r$. In Figure 3 and Figure 4, a fixed number of r=5 equal canonical correlations $\varrho_{i}\in \left\{0.1,0.2,0.5,0.7,0.9\right\},i=1,\ldots ,r$ is considered. When all canonical correlations are equal, then, due to the central limit theorem, the distribution of the information density i(ξ;η) converges to a Gaussian distribution as r→∞. Figure 5 and Figure 6 show for r∈{5,10,20,40} and equal canonical correlations $\varrho_{i}=0.2,i=1,2,\ldots ,r$ the PDF and CDF of the information density together with corresponding Gaussian approximations. The approximations are obtained by considering Gaussian distributions, which have the same variance as the information density i(ξ;η). Recall that the variance of the information density is given by (11), i.e., by the sum of the squared canonical correlations. The illustrations show that only for a high number of equal canonical correlations the distribution of the information density becomes approximately Gaussian.

**Different canonical correlations.** To illustrate the case with different canonical correlations, let us consider two more examples.

(i) First, assume that the random vectors $\xi =(\xi_{1},\xi_{2},\ldots ,\xi_{p})$ and $\eta =(\eta_{1},\eta_{2},\ldots ,\eta_{q})$ have equal dimensions, i.e., p=q, and are related by

$$\begin{array}{c} \left(\eta_{1},\eta_{2},\ldots ,\eta_{p}\right)=\left(\xi_{1}+\zeta_{1},\xi_{2}+\zeta_{2},\ldots ,\xi_{p}+\zeta_{p}\right), \end{array}$$

where $\xi =(\xi_{1},\xi_{2},\ldots ,\xi_{p})$ and $\zeta =(\zeta_{1},\zeta_{2},\ldots ,\zeta_{p})$ are zero mean Gaussian random vectors, independent of each other and with covariance matrices

$$\begin{array}{c} R_{\xi }={\left(\rho^{\left|i-j\right|}\right)}_{i,j=1}^{p}\;\;\mathrm{and}\;R_{\zeta }=\sigma_{z}^{2}I_{p}, \end{array}$$

for parameters 0<|ρ|<1 and $\sigma_{z}^{2}>0$, where $I_{p}$ denotes the identity matrix of dimension p×p. The covariance matrix of the Gaussian random vector $\left(\xi_{1},\xi_{2},\ldots ,\xi_{p},\eta_{1},\eta_{2},\ldots ,\eta_{p}\right)$ is the basis of the canonical correlation analysis and is given by

$$\left(\begin{array}{cc} R_{\xi } & R_{\xi \eta }\\ R_{\xi \eta } & R_{\eta } \end{array}\right)=\left(\begin{array}{cc} R_{\xi } & R_{\xi }\\ R_{\xi } & R_{\xi }+R_{\zeta } \end{array}\right)\;.$$

The specified situation corresponds to a discrete-time additive noise channel, where a stationary first-order Markov-Gaussian input process is corrupted by a stationary additive white Gaussian noise process. In this setting, a block of *p* consecutive input and output symbols is considered.

For given parameter values ρ and $\sigma_{z}^{2}$, the canonical correlations can be calculated numerically with the method described in Section 3. However, the example at hand even allows the derivation of explicit formulas for the canonical correlations. Evaluating the approach in Section 3 analytically yields

(70) $$\begin{array}{c} \varrho_{i}\left(\rho ,\sigma_{z}^{2}\right)=\sqrt{\frac{\lambda_{i}}{\lambda_{i}+\sigma_{z}^{2}}}\;\mathrm{with}\;\lambda_{i}=\frac{1-\rho^{2}}{1-2\rho \cos\left(\theta_{i}\right)+\rho^{2}},\;i=1,2,\ldots ,r=p, \end{array}$$

where $\theta_{1},\theta_{2},\ldots ,\theta_{r}$ are the zeros of the function

$$\begin{array}{c} g\left(\theta \right)=\sin\left((r+1)\theta \right)-2\rho \sin\left(r\theta \right)+\rho^{2}\sin\left((r-1)\theta \right),\;\theta \in \left(0,\pi \right). \end{array}$$

In this representation, $\lambda_{1},\lambda_{2},\ldots ,\lambda_{r}$ denote the eigenvalues of the covariance matrix $R_{\xi }={\left(\rho^{\left|i-j\right|}\right)}_{i,j=1}^{p}$ derived in [29], Sec. 5.3.

**Figure 7:**
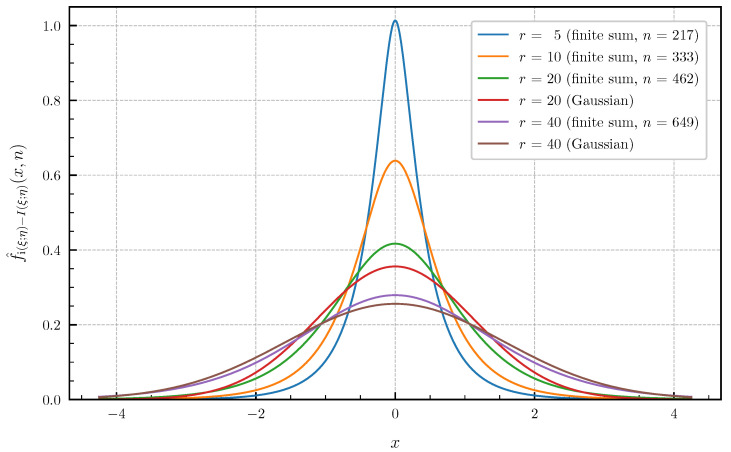
Approximated PDF ${\hat{f}}_{i(\xi ;\eta )-I(\xi ;\eta )}\left(\cdot ,n\right)$ for r∈{5,10,20,40} canonical correlations $\varrho_{i}\left(\rho ,\sigma_{z}^{2}\right)$ given in (70) for ρ=0.9 and $\sigma_{z}^{2}=10$ (approximation error $<1\;\times \;10^{-3}$) vs. Gaussian approximation (r∈{20,40}).

**Figure 8:**
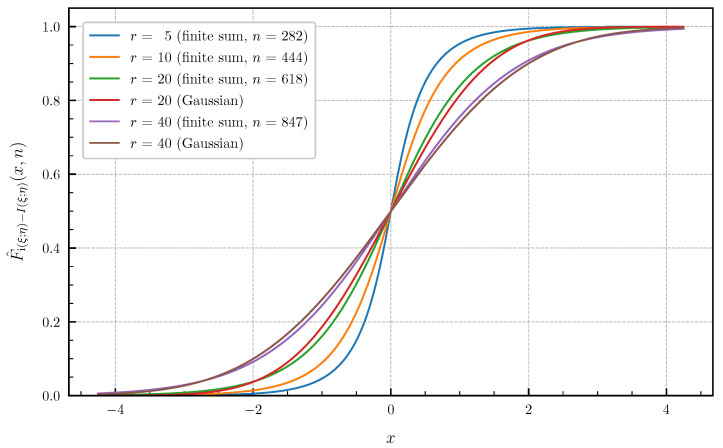
Approximated CDF ${\hat{F}}_{i(\xi ;\eta )-I(\xi ;\eta )}\left(\cdot ,n\right)$ for r∈{5,10,20,40} canonical correlations $\varrho_{i}\left(\rho ,\sigma_{z}^{2}\right)$ given in (70) for ρ=0.9 and $\sigma_{z}^{2}=10$ (approximation error $<1\;\times \;10^{-3}$) vs. Gaussian approximation (r∈{20,40}).

As numerical examples Figure 7 and Figure 8 show, the approximated PDF ${\hat{f}}_{i(\xi ;\eta )-I(\xi ;\eta )}\left(\cdot ,n\right)$ and CDF ${\hat{F}}_{i(\xi ;\eta )-I(\xi ;\eta )}\left(\cdot ,n\right)$ for p=r∈{5,10,20,40} and the parameter values ρ=0.9 and $\sigma_{z}^{2}=10$ using the finite sums (62) and (64). The bounds of the approximation error given in Theorem 4 are chosen $<1\;\times \;10^{-3}$ to obtain a high precision of the plotted curves. The number *n* of summands required in (62) and (64) to achieve these error bounds for r∈{5,10,20,40} is equal to n∈{217,333,462,649} for the PDF and n∈{282,444,618,847} for the CDF. For this example, the distribution of the information density i(ξ;η) converges to a Gaussian distribution as r→∞. However, Figure 7 and Figure 8 show that, even for r=40, there is still a significant gap between the exact distribution and the corresponding Gaussian approximation.

(ii) As a second example with different canonical correlations, let us consider the sequence $\{\varrho_{1}\left(T\right),\varrho_{2}\left(T\right),$$\ldots ,\varrho_{r}\left(T\right)\}$ with

(71) $$\begin{array}{c} \varrho_{i}\left(T\right)=\sqrt{\frac{T^{2}}{T^{2}+\pi {\left(i-\frac{1}{2}\right)}^{2}}},\;i=1,2,\ldots ,r. \end{array}$$

**Figure 9:**
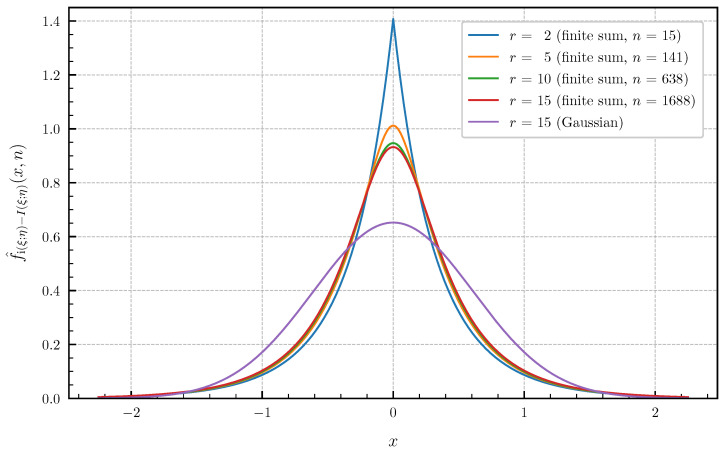
Approximated PDF ${\hat{f}}_{i(\xi ;\eta )-I(\xi ;\eta )}\left(\cdot ,n\right)$ for r∈{2,5,10,15} canonical correlations $\varrho_{i}\left(T\right)$ given in (71) for T=1 (approximation error $<1\;\times \;10^{-2}$) vs. Gaussian approximation (r=15).

**Figure 10:**
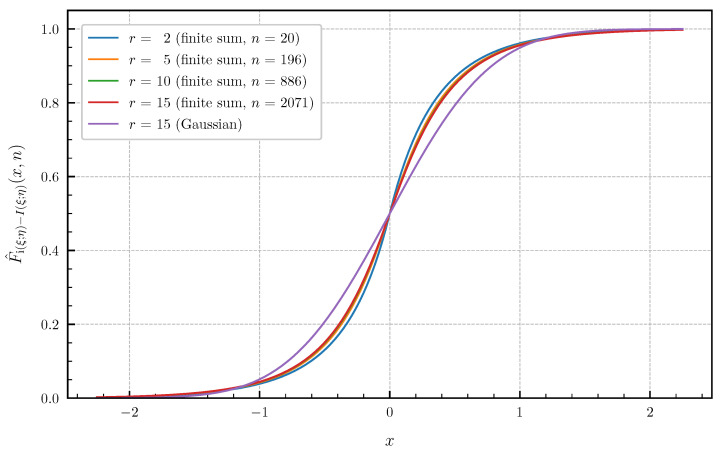
Approximated CDF ${\hat{F}}_{i(\xi ;\eta )-I(\xi ;\eta )}\left(\cdot ,n\right)$ for r∈{2,5,10,15} canonical correlations $\varrho_{i}\left(T\right)$ given in (71) for T=1 (approximation error $<\;1\times \;10^{-2}$) vs. Gaussian approximation (r=15).

These canonical correlations are related to the information density of a continuous-time additive white Gaussian noise channel confined to a finite time interval [0,T] with a Brownian motion as input signal (see, e.g., Huffmann [30], Sec. 8.1 for more details). Figure 9 and Figure 10 show the approximated PDF ${\hat{f}}_{i(\xi ;\eta )-I(\xi ;\eta )}\left(\cdot ,n\right)$ and CDF ${\hat{F}}_{i(\xi ;\eta )-I(\xi ;\eta )}\left(\cdot ,n\right)$ for r∈{2,5,10,15} and T=1 using the finite sums (62) and (64). The bounds of the approximation error given in Theorem 4 are chosen $<\;1\;\times \;10^{-2}$ such that there are no differences visible in the plotted curves by further lowering the approximation error. The number *n* of summands required in (62) and (64) to achieve these error bounds for r∈{2,5,10,15} is equal to n∈{15,141,638,1688} for the PDF and n∈{20,196,886,2071} for the CDF. Choosing *r* larger than 15 for the canonical correlations (71) with T=1 does not result in visible changes of the PDF and CDF compared to r=15. This demonstrates, together with Figure 9 and Figure 10, that a Gaussian approximation is not valid for this example, even if r→∞.

Indeed, from [8], Th. 9.6.1 and the comment above Eq. (9.6.45) in [8], one can conclude that, whenever the canonical correlations satisfy

$$\begin{array}{c} \lim_{r\to \infty }\;\sum_{i=1}^{r}\varrho_{i}^{2}<\;\infty , \end{array}$$

then the distribution of the information density is *not* Gaussian.

## 7. Summary of Contributions

We derived series representations of the PDF and CDF of the information density for arbitrary Gaussian random vectors as well as a general formula for the central moments using canonical correlation analysis. We provided simplified and closed-form expressions for important special cases, in particular when all canonical correlations are equal, and derived recurrence formulas and uniform error bounds for finite sum approximations of the general series representations. These approximations and recurrence formulas are suitable for efficient and arbitrarily accurate numerical calculations, where the approximation error can be easily controlled with the derived error bounds. Moreover, we provided examples showing the (in)validity of approximating the information density with a Gaussian random variable.

## Data Availability

An implementation in Python allowing efficient numerical calculations related to the main results of the paper is publicly available on GitLab: https://gitlab.com/infth/information-density (accessed on 24 June 2022).

## References

[1] Han T.S., Verdú S. (1993). Approximation Theory of Output Statistics. IEEE Trans. Inf. Theory.

[2] Han T.S. (2003). Information-Spectrum Methods in Information Theory.

[3] Shannon C.E. (1959). Probability of Error for Optimal Codes in a Gaussian Channel. Bell Syst. Tech. J..

[4] Dobrushin R.L. (1961). Mathematical Problems in the Shannon Theory of Optimal Coding of Information. Proceedings of the Fourth Berkeley Symposium on Mathematical Statistics and Probability.

[5] Strassen V. (1964). Asymptotische Abschätzungen in Shannons Informationstheorie. Transactions of the Third Prague Conference on Information Theory, Statistical Decision Functions, Random Processes (Held 1962).

[6] Polyanskiy Y., Poor H.V., Verdú S. (2010). Channel Coding Rate in the Finite Blocklength Regime. IEEE Trans. Inf. Theory.

[7] Durisi G., Koch T., Popovski P. (2016). Toward Massive, Ultrareliable, and Low-Latency Wireless Communication With Short Packets. Proc. IEEE.

[8] Pinsker M.S. (1964). Information and Information Stability of Random Variables and Processes.

[9] Olver F.W.J., Lozier D.W., Boisvert R.F., Clark C.W. (2010). NIST Handbook of Mathematical Functions.

[10] Mathai A.M. (1982). Storage Capacity of a Dam With Gamma Type Inputs. Ann. Inst. Stat. Math..

[11] Grad A., Solomon H. (1955). Distribution of Quadratic Forms and Some Applications. Ann. Math. Stat..

[12] Kotz S., Johnson N.L., Boyd D.W. (1967). Series Representations of Distributions of Quadratic Forms in Normal Variables. I. Central Case. Ann. Math. Stat..

[13] Huffmann J.E.W., Mittelbach M. (2022). On the Distribution of the Information Density of Gaussian Random Vectors: Explicit Formulas and Tight Approximations. Entropy.

[14] Simon M.K. (2006). Probability Distributions Involving Gaussian Random Variables: A Handbook for Engineers and Scientists.

[15] Laneman J.N. On the Distribution of Mutual Information. Proceedings of the Workshop Information Theory and Its Applications (ITA).

[16] Wu P., Jindal N. (2011). Coding Versus ARQ in Fading Channels: How Reliable Should the PHY Be?. IEEE Trans. Commun..

[17] Buckingham D., Valenti M.C. The Information-Outage Probability of Finite-Length Codes Over AWGN Channels. Proceedings of the 42nd Annual Conference on Information Sciences and Systems (CISS).

[18] Hotelling H. (1936). Relations Between Two Sets of Variates. Biometrika.

[19] Gelfand I.M., Yaglom A.M. (1959). Calculation of the Amount of Information About a Random Function Contained in Another Such Function. AMS Translations, Series 2.

[20] Härdle W.K., Simar L. (2015). Applied Multivariate Statistical Analysis.

[21] Koch I. (2014). Analysis of Multivariate and High-Dimensional Data.

[22] Timm N.H. (2002). Applied Multivariate Analysis.

[23] Ibragimov I.A., Rozanov Y.A. (1970). On the Connection Between Two Characteristics of Dependence of Gaussian Random Vectors. Theory Probab. Appl..

[24] Gradshteyn I.S., Ryzhik I.M. (2007). Table of Integrals, Series, and Products.

[25] Prudnikov A.P., Brychov Y.A., Marichev O.I. (1986). Integrals and Series, Volume 2: Special Functions.

[26] Huffmann J.E.W., Mittelbach M. (2021). Efficient Python Implementation to Numerically Calculate PDF, CDF, and Moments of the Information Density of Gaussian Random Vectors. https://gitlab.com/infth/information-density.

[27] Moschopoulos P.G. (1985). The Distribution of the Sum of Independent Gamma Random Variables. Ann. Inst. Stat. Math..

[28] Comtet L. (1974). Advanced Combinatorics: The Art of Finite and Infinite Expansions.

[29] Grenander U., Szegö G. (1958). Toeplitz Forms and Their Applications.

[30] Huffmann J.E.W. (2021). Canonical Correlation and the Calculation of Information Measures for Infinite-Dimensional Distributions. Diploma Thesis.

